# pZMO7-Derived shuttle vectors for heterologous protein expression and proteomic applications in the ethanol-producing bacterium *Zymomonas mobilis*

**DOI:** 10.1186/1471-2180-14-68

**Published:** 2014-03-15

**Authors:** Lok Yan So, Wen-yang Chen, Donnabella C Lacap-Bugler, Myriam Seemann, Rory M Watt

**Affiliations:** 1 Oral Biosciences Faculty of Dentistry, The University of Hong Kong, 34 Hospital Road, Sai Ying Pun, Hong Kong; 2 Oral Diagnosis and Polyclinics, Faculty of Dentistry, The University of Hong Kong, Prince Philip Dental Hospital, 34 Hospital Road, Sai Ying Pun, Hong Kong; 3 Université de Strasbourg, CNRS UMR 7177, Institut Le Bel, 4 rue Blaise Pascal, CS 90032, Strasbourg 67081Cedex, France; 4 Present Address: Department of Applied Biology and Chemical Technology, The Hong Kong Polytechnic University, Hung Hom, Kowloon, Hong Kong

**Keywords:** *Zymomonas mobilis*, Plasmid, Shuttle vector, Replication, Quantitative PCR, Proteomics, Affinity purification, Microbial biotechnology

## Abstract

**Background:**

The ethanol-producing bacterium *Zymomonas mobilis* has attracted considerable scientific and commercial interest due to its exceptional physiological properties. Shuttle vectors derived from native plasmids have previously been successfully used for heterologous gene expression in this bacterium for a variety of purposes, most notably for metabolic engineering applications.

**Results:**

A quantitative PCR (qPCR) approach was used to determine the copy numbers of two endogenous double stranded DNA plasmids: pZMO1A (1,647 bp) and pZMO7 (pZA1003; 4,551 bp) within the NCIMB 11163 strain of *Z. mobilis*. Data indicated pZMO1A and pZMO7 were present at *ca*. 3-5 and *ca*. 1-2 copies per cell, respectively. A *ca.* 1,900 bp fragment from plasmid pZMO7 was used to construct two *Escherichia coli* – *Z. mobilis* shuttle vectors (pZ7C and pZ7-184). The intracellular stabilities and copy numbers of pZ7C and pZ7-184 were characterized within the NCIMB 11163, ATCC 29191 and (ATCC 10988-derived) CU1 Rif2 strains of *Z. mobilis*. Both shuttle vectors could be stably maintained within the ATCC 29191 strain (*ca*. 20-40 copies per cell), and the CU1 Rif2 strain (*ca*. 2-3 copies per cell), for more than 50 generations in the absence of an antibiotic selectable marker. A selectable marker was required for shuttle vector maintenance in the parental NCIMB 11163 strain; most probably due to competition for replication with the endogenous pZMO7 plasmid molecules. N-terminal glutathione S-transferase (GST)-fusions of four endogenous proteins, namely the acyl-carrier protein (AcpP); 2-dehydro-3-deoxyphosphooctonate aldolase (KdsA); DNA polymerase III chi subunit (HolC); and the RNA chaperone protein Hfq; were successfully expressed from pZ7C-derived shuttle vectors, and their protein-protein binding interactions were analyzed in *Z. mobilis* ATCC 29191. Using this approach, proteins that co-purified with AcpP and KdsA were identified.

**Conclusions:**

We show that a shuttle vector-based protein affinity ‘pull-down’ approach can be used to probe protein interaction networks in *Z. mobilis* cells. Our results demonstrate that protein expression plasmids derived from pZMO7 have significant potential for use in future biological or biotechnological applications within *Z. mobilis*.

## Background

*Zymomonas mobilis* is a Gram-negative facultative anaerobic bacterium, which has attracted significant interest over recent years for its use in the industrial-scale production of ‘bioethanol’ [1-5]. This microorganism is able to ferment glucose, fructose or sucrose to ethanol, with extremely high molecular efficiencies and minimum accompanying levels of biomass formation. As a ‘generally regarded as safe’ (GRAS) microorganism, *Z. mobilis* has also been used for a variety of other biotechnological purposes, such as the production of levan (polyfructan) [6,7] or amino acids [8]. Over the past 20 years or so, significant effort has been spent on genetically ‘engineering’ its metabolic capabilities and physiological activities. These have largely focused on extending its limited substrate range, enabling it to utilize carbohydrates that are abundant in lignocellulosic feedstocks [2,4,5,9-12]. Genetic engineering applications in *Z. mobilis* have commonly utilized plasmid vectors housing heterologous genes encoding proteins with the desired functionalities [12].

Cloning vectors that are routinely used in *Escherichia coli*, such as those derived from pBR322 or pUC18, cannot be stably-maintained in *Z. mobilis*[12]. On the other hand, several types of bacterial broad-host range plasmids are able to replicate in *Z. mobilis* cells (e.g. derivatives of pBBR1MCS, RSF1010 and the incW R plasmid Sa), and have been used for a variety of heterologous gene expression applications. However, they are prone to structural (genetic) instability, and their relatively large size constrains gene cloning strategies [12-15]. Consequently, the most common approach for heterologous gene expression in *Z. mobilis* has involved *E. coli* – *Z. mobilis* shuttle vectors; which incorporate replicons from *E. coli* plasmids, as well as those from native plasmids isolated from various *Z. mobilis* strains [12,13,16-22].

Four native plasmids from *Z. mobilis* ATCC 10988 have been used as the basis for the construction of shuttle vectors: pZMO1 (1,651 bp) [21], pZMO2 (1,669 bp) [20-22], pZM2 (pZMO3; 2,749 bp) [16,23,24] and pZM3 (pZMOB05; 4,023 bp) [17,25]. Shuttle vectors have also been constructed from native plasmids isolated from other *Z. mobilis* strains, such as pNSW301 from the ZM6100 strain [26]; pZMPI from the *Z. mobilis* PROM Al strain [27] and pZA2 from the NCIMB 8827 strain [19]. Of these, the pZM2 (pZMO3) plasmid has been used most extensively for the construction of expression plasmids for physiological investigations or industrial applications in *Z. mobilis*, e.g. [9,10,12,16,28,29]. Most notably, the pZM2-derived pZB5 plasmid, which houses four genes involved in pentose sugar metabolism, was used to broaden the substrate range of the CP4 strain, enabling it to utilize xylose for the bioproduction of ethanol [9,10]. Plasmids derived from pZM2 have also been used to express green fluorescent protein reporters [30]; to produce proteins of biotechnological interest such as the InaZ ice-nucleation protein [28]; to express fungal carotenoid biosynthetic proteins to direct the production of beta-carotene [31]; and to produce and secrete cellulolytic enzymes to facilitate the utilization of lignocellulosic biomass [29].

In microbial cells, proteins often function within hetero-multimeric complexes, or have activities that are directly modulated by protein-protein interactions [32,33]. Approaches involving various combinations of affinity chromatography and mass spectrometry have previously been employed to establish large-scale protein interaction networks, known as ‘interactomes’, within prokaryotic and eukaryotic microorganisms [34,35]. However, to the best of our knowledge, protein-protein interaction analyses have never been performed in *Z. mobilis* or a related alphaproteobacterial species.

**Figure 1:**
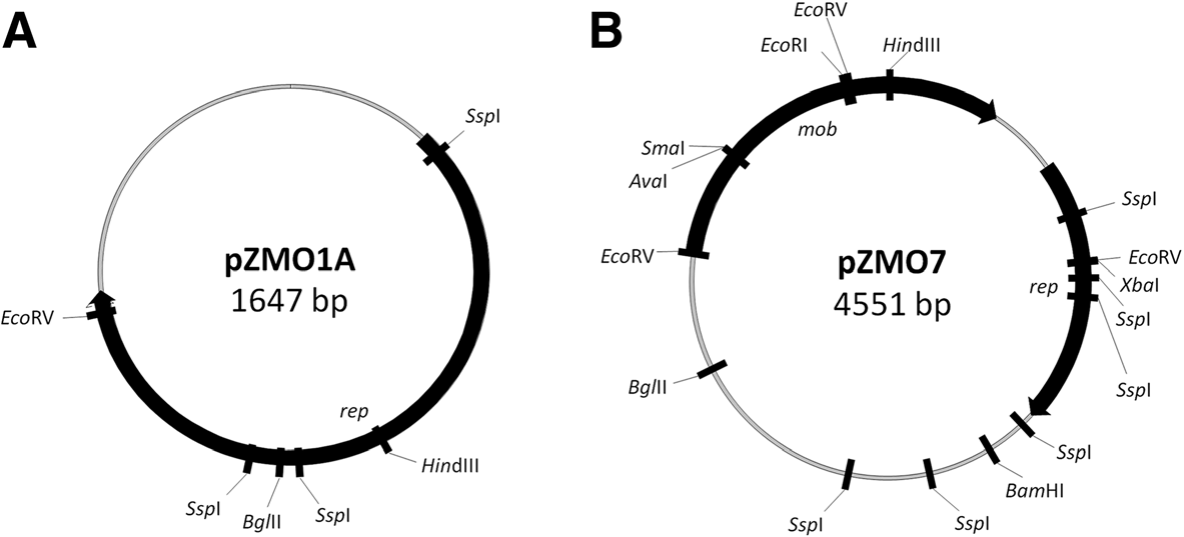
**Restriction maps of two native plasmids from Z. mobilis NCIMB 11163. (A)** pZMO1A **(B)** pZMO7.

The genome sequence for *Z. mobilis* NCIMB 11163 was recently published [36]. This included the sequences of three endogenous plasmids: p11163_1 (deposited as pZA1001; 53,380 bp), p11163_2 (pZA1002; 40,818 bp) and p11163_3 (pZA1003; 4,551 bp). This was consistent with results from our own *Z. mobilis* plasmid sequencing efforts, in which we had determined the sequences of the two smallest plasmids from NCIMB 11163: pZMO1A (1,647 bp) and pZMO7 (4,551 bp) (Figure 1) [37]. The sequences of pZMO7 and p11163_3 (pZA1003) are identical, and they correspond to the same plasmid. Due to its relatively small size and genetic composition (see below), we hypothesized that pZMO7 may be suitable for shuttle vector development.

The aim of this study was to develop an *Escherichia coli-Z. mobilis* shuttle-vector system based on pZMO7, and determine its potential for heterologous protein expression and proteomic applications within *Z. mobilis*. To achieve this, we constructed a shuttle vector backbone (pZ7C) that contained a *ca*. 1,900 bp replicon fragment from pZMO7. We determined the stability and copy number of pZ7C within three different *Z. mobilis* strain lineages: NCIMB 11163; CU1 Rif2 [20]; and the ATCC 29191 centrotype strain [1]; under selective and non-selective conditions. As a proof of principle, we expressed a variety of glutathione S-transferase (GST)-fusion proteins from pZ7C-derived shuttle vectors established in *Z. mobilis* ATCC 29191, and analyzed their intracellular protein-protein binding interactions. Our results demonstrate the utility of pZMO7-derived shuttle vectors for biological applications in *Z. mobilis*.

## Methods

### Bacterial strains and culture conditions

Bacterial strains are listed in Table 1. Unless otherwise stated, liquid cultures of *Z. mobilis* cells were grown semi-aerobically in Rich Medium (RM) [38,39] without agitation at 30°C; in Falcon tubes (BD Biosciences), or Duran laboratory glass bottles (Schott AG), with caps that were fitted, but not air-tight; to allow limited gaseous exchange. Optical density measurements at 600 nm (OD_600nm_) were determined using a Beckman DU 530 Life Science UV/Vis spectrophotometer (Beckman Coulter Inc., USA). *E. coli* strains were grown aerobically in Luria Broth (LB) at 37°C. For agar plate preparation, 1.5% w/v agar was added. Plates were incubated aerobically at 30°C for *Z. mobilis* strains, or 37°C for *E. coli* strains. Antibiotics were used at the following concentrations: 100 μg/ml chloramphenicol (Cm) for *Z. mobilis*; 100 μg/ml ampicillin (Amp), 30 μg/ml Cm and 10 μg/ml tetracycline (Tc) for *E. coli*.

**Table 1:** Strains and plasmids used in this study

**Bacterial strain or plasmid**	**Genotype, relevant plasmid markers and/or other characteristics**	**Source/reference**
*Escherichia coli*		
DH10B	F^-^*mcrA* ∆(*mrr-hsdRMS-mcrBC*) Ф80d*lacZ*∆M15 ∆*lacX74 deoR recA1 endA1 araD139* ∆(*ara-leu*)7649 *galU galK rspL nupG*	Invitrogen
BL21 (DE3)	*E. coli B dcm ompT hsdS(rB-mB-) gal*	Stratagene
*Zymomonas mobilis subsp. mobilis*		
NCIMB 11163	Wild-type strain	NCIMB, C. Drainas
CU1 Rif2	Mutant strain of ATCC 10988	C. Drainas [20]
ATCC 29191	Wild-type strain, DSM 3580, z6, NCIMB 11199	DSMZ
Plasmids		
pACYC184	*cm*^*r*^, *tet*^*r*^, *ori* (p15A)	NEB
pUC18	*amp*^*r*^, *ori* (pMB1)	Stratagene
pGEX4T1	*amp*^*r*^, *ori* (pMB1), *lacI,* P_tac_-*gst*-Term	GE Healthcare
Cm-pUC18	*cm*^*r*^, *ori* (pMB1)	This study
pUCZM-1	*cm*^*r*^, *rep* (pZMO1A), *ori* (pZMO1A), *ori* (pMB1)	This study
pUCZM-3	*cm*^*r*^, *rep* (pZMO7), *ori* (pZMO7), *ori* (pMB1)	This study
pZ7-184	*cm*^*r*^, *rep* (pZMO7), *ori* (pZMO7), *ori* (p15A)	This study
pZ7C	*cm*^*r*^, *rep* (pZMO7), *ori* (pZMO7), *ori* (pMB1)	This study
pZ7-GST	*cm*^*r*^, *rep* (pZMO7), *ori* (pZMO7), P_tac_-*gst*-Term, *ori* (pMB1)	This study
pZ7-GST-acpP	*cm*^*r*^, *rep* (pZMO7), *ori* (pZMO7), P_tac_-*gst*-*acpP*-Term, *ori* (pMB1)	This study
pZ7-GST-dnaJ	*cm*^*r*^, *rep* (pZMO7), *ori* (pZMO7), P_tac_-*gst*-*dnaJ*-Term, *ori* (pMB1)	This study
pZ7-GST-hfq	*cm*^*r*^, *rep* (pZMO7), *ori* (pZMO7), P_tac_-*gst*-*hfq*-Term, *ori* (pMB1)	This study
pZ7-GST-holC	*cm*^*r*^, *rep* (pZMO7), *ori* (pZMO7), P_tac_-*gst*-*holC*-Term, *ori* (pMB1)	This study
pZ7-GST-kdsA	*cm*^*r*^, *rep* (pZMO7), *ori* (pZMO7), P_tac_-*gst*-*kdsA*-Term, *ori* (pMB1)	This study
ppk2-TOPO	*amp*^*r*^*,kan*^*r*^*, ori *(ColE1), P_lac_-*lacZα*, *ori *(f1)	This study

### DNA amplification and manipulations

Plasmid DNA was recovered from *E. coli* DH10B or *Z. mobilis* cultures using QiaPrep Spin Miniprep kits (Qiagen, CA, USA). The sequences of all primers are shown in Additional file 1. PCR products were purified using QIAquick PCR purification kits (Qiagen, CA, USA) or gel-purified using QIAquick Gel Extraction kits (Qiagen, CA, USA) following the manufacturers’ protocols. All cloned PCR-amplified inserts and junctions between ligated DNA fragments were sequenced bidirectionally to confirm the integrity of all plasmid constructs (Applied Biosystems 3730xl DNA Analyzer, BGI Hong Kong Ltd.).

### Transformation of DNA into *Z. mobilis* cells

Plasmid DNA (1.5 μl, *ca*. 400 ng/μl) was transformed into *Z. mobilis* competent cells (100 μl, freshly prepared from single colonies) as previously described by Liang *et al*. [40]; using a BioRad MicroPulser (Bio-Rad, USA) with 1 mm gap electroporation cuvettes (4-5.6 ms pulse duration; 1.8 kV pulse). Transformed cells were recovered in RM medium (1 ml), incubating semi-aerobically at 30°C for 2-3 hours, before plating onto RM agar containing 100 μg/ml Cm for clone selection.

### Construction of *Z. mobilis* NCIMB 11163 native plasmid library

**Figure 2:**
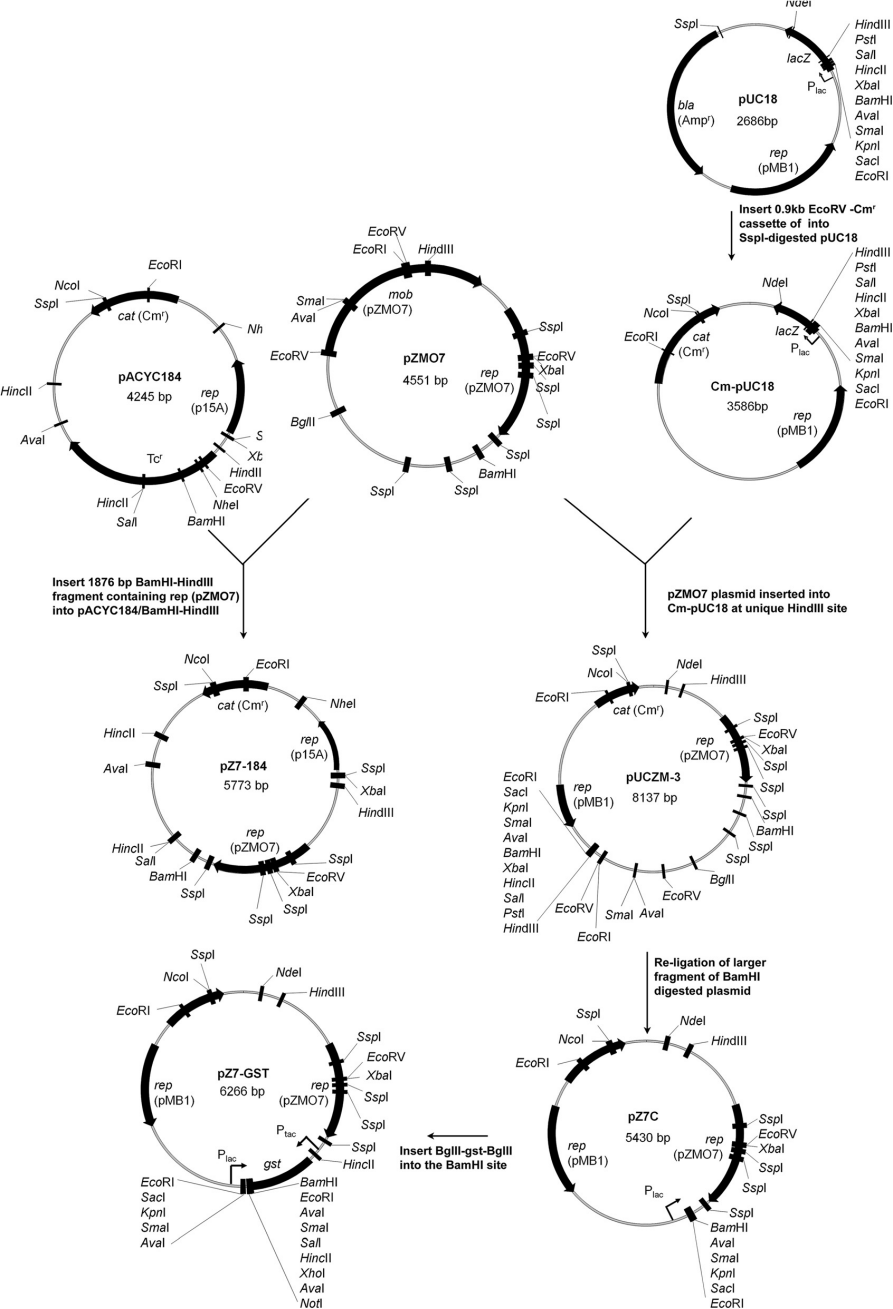
Schematic diagram outlining the construction of the pZMO7-derived shuttle vectors used in this study.

A chloramphenicol resistance (*Cm*^*r*^) cassette was PCR amplified from plasmid pLysS (Novagen, EMD Millipore, Germany) using the Cm-F and Cm-R primers, digested with *Eco*RV and then blunt-end ligated to *Ssp*I-digested pUC18 plasmid (Stratagene, Agilent Technologies, USA) to produce Cm-pUC18, thereby inactivating the *bla* (*Amp*^*r*^) gene. Purified *Z. mobilis* NCIMB 11163 endogenous plasmid DNA was digested with *Hin*dIII (New England Biolabs (NEB), USA), purified (QIAquick PCR purification kit), ligated into *Hind*III-linearized Cm-pUC18 (Figure 2), and electroporated into *E. coli* DH10B (Invitrogen, Life Technologies, USA). Colonies were screened for presence of an intact *Cm*^*r*^ cassette by streaking onto LB + Cm plates, using LB + Amp for negative selection. Plasmid DNA was purified from Cm-resistant transformant colonies, whose inserts were sequenced bidirectionally using M13 primers, followed by a ‘primer walking’ approach, giving 2-3 times sequence coverage. Plasmids pUCZM-1 and pUCZM-3 from this library respectively contained the entire pZMO1A and pZMO7 plasmids in a *Hind*III-linearized form (see Table 1).

### Construction of pZMO7-derived expression vectors

The 1,876 bp *Hin*dIII*/Bam*HI fragment from pUCZM-3 was ligated into plasmid pACYC-184 (NEB) forming the plasmid pZ7-184 (Figure 2). Plasmid pUCZM-3 was digested with *Bam*HI, and the resultant 5,430 bp fragment was purified and self-ligated to form plasmid pZ7C. The fragment containing the P_tac_ promoter, glutathione S-transferase (*gst*) gene, multiple cloning site and downstream terminator (Term) sequence (P_tac_–*gst*–MCS-Term expression cassette) was PCR amplified from plasmid pGEX4T1 (GE Healthcare, Life Sciences, USA) using the Ptac-F and Ttac-R primers, digested with *Bgl*II and inserted into the *Bam*HI site of pZ7C plasmid to produce pZ7-GST (Figure 2). The acyl-carrier protein (*acpP*, ZZ6_0066); chaperone protein DnaJ (ZZ6_0618), RNA chaperone protein Hfq (ZZ6_0899), DNA polymerase III chi subunit (*holC*, ZZ6_0042) and 2-dehydro-3-deoxyphosphooctonate aldolase protein (*kdsA*, ZZ6_1604) genes were PCR amplified from *Z. mobilis* ATCC 29191. The genes were respectively cloned into pZ7-GST via *Bam*HI/*Xho*I to form the pZ7-GST-acpP, pZ7-GST-dnaJ, pZ7-GST-hfq, pZ7-GST-holC and pZ7-GST-kdsA plasmids, respectively. All plasmid constructs were verified by sequence analysis.

### Determination of plasmid stability in *Z. mobilis*

Plasmid stability was determined following the method described by Conway *et al*. [41]. Cultures of freshly-transformed *Z. mobilis* cells (inoculated from single colonies) were incubated in RM media containing 100 μg/ml Cm (10 ml) without agitation at 30°C for *ca*. 24 hours. Aliquots (100 μl) were expanded 1:100 into fresh RM media lacking Cm (10 ml), and were cultured at 30°C for 24 hours without agitation. This iterative sub-culturing process was repeated every 24 hours, for 5 consecutive days. Aliquots were withdrawn daily for: 1) plasmid isolation and analysis by agarose gel electrophoresis (after *Hind*III digestion); 2) quantitative PCR analysis (see below).

### Determination of relative amounts of pZMO1A and pZMO7 plasmids using a gel-based approach

‘Stabs’ from single colonies of freshly-plated *Z. mobilis* NCIMB 11163 with minimal passage were grown semi-aerobically without agitation in RM media (15 ml, 50 ml capped Falcon tubes) at 30°C for *ca.* 24 hours until OD_600nm_*ca.* 0.6. Plasmid DNA was extracted (QIAprep spin miniprep kit; Qiagen), and an aliquot was digested (*Hin*dIII) to linearize the pZMO1A and pZMO7 plasmids present. Aliquots of undigested and *Hin*dIII-digested plasmid DNA were analyzed on 0.8% agarose/TAE gels using ethidium bromide staining on a Bio-Rad ChemiDoc XRS instrument (Bio-Rad, USA). Band intensities on negative scanned gel images were quantified using Quantity One software (BioRad) to determine the relative proportions of pZMO1A and pZMO7 plasmids present.

### Extraction of plasmid and chromosomal DNA for quantitative real time PCR analysis

The cell lysis and crude DNA extraction procedure used was based on the method described by Skulj *et al.*[42]. Freshly-inoculated cultures of recombinant or wild type *Z. mobilis* strains were incubated semi-aerobically without agitation at 30°C to OD_600nm_ of *ca.* 0.25 in RM media (4 ml, 15 ml capped Falcon tubes) with/without 100 μl/ml chloramphenicol (as indicated in the text). After centrifugation (4,000 x g, 10 mins, 2-4°C), cell pellets were washed with ice cold EB buffer [Tris-HCl (10 mM) pH 8.5], then resuspended in EB buffer (100 μl), heated in boiling water for 30 mins, before allowing to cool gradually to room temperature. The supernatant obtained after centrifugation (14,000 x g, 10 min) was used directly as template for quantitative (real time) PCR analyses.

### Quantitative real time PCR analysis

Plasmid copy numbers were determined by quantitative real time PCR (qPCR) using a relative quantification approach, based on the procedure described by Skulj *et al.*[42]. qPCR was performed in 20 μl reaction mixtures in MicroAmp optical 48-well reaction plates, using the Fast SYBR Green PCR Master Mix reagent (Applied Biosystems, CA, USA) on a StepOnePlus Real-Time PCR system (Applied Biosystems, CA, USA) controlled by StepOne Software Version 2.0 (Applied Biosystems). Primers were designed using Primer Express Software Version 3.0 (Applied Biosystems; see Additional file 1 for qPCR primer sequences). Plasmid DNA concentrations were determined using a Nanodrop 2000 spectrophotometer (Thermo Scientific, DE, USA). Serial dilutions of the pUCZM-1 and pUCZM-3 plasmids were used to create standard curves for quantifying pZMO1A and pZMO7 plasmid concentrations. A pCR2.1 TOPO vector containing the PCR-amplified polyphosphate kinase 2 (*ppk2,* ZZ6_0566) gene from *Z. mobilis* ATCC 29191 (ppk2-TOPO) was similarly used to construct a standard curve for *Z. mobilis* chromosome copy number determination. Concentrations of chromosome molecules, native plasmids and recombinant plasmids were individually quantified by qPCR within aliquots from the same freshly-prepared cell lysate supernatants prepared from wild-type or transformed *Z. mobilis* strain cultures (as described above). The (relative) plasmid copy numbers (PCNs) in each sample were calculated by dividing the concentration of the respective plasmid molecules by the concentration of chromosome molecules. All qPCR experiments were performed in duplicate, with at least two independent biological replicates.

### Analysis of pZ7C plasmid-based Glutathione S-Transferase (GST) and GST fusion protein expression in *E. coli and Z. mobilis*

Freshly-transformed starter cultures of recombinant *E. coli* BL21 (DE3) strains containing the pZ7-GST, pZ7-GST-acpP, pZ7-GST-dnaJ, pZ7-GST-hfq, pZ7-GST-holC or pZ7-GST-kdsA plasmids in LB media containing 30 μg/ml Cm were expanded 1:50 into fresh LB containing 30 μg/ml Cm (800 ml) and grown aerobically with shaking (37°C) until OD_600nm_ of *ca*. 1.0. Cultures were chilled in ice-water, and cell pellets were collected by centrifugation (4,000 x g, 10 mins 2-4°C), washed with 10% aqueous glycerol, then resuspended in 20 ml ice-cold binding buffer (25 mM Tris-HCl pH 7.4, 200 mM NaCl, 1 mM EDTA, 1.5 mM beta-mercaptoethanol). Cells were lysed by sonication with ice-cooling (Sonics Vibra-Cell, 40% amplitude; 5 cycles of: 3 s pulse-on, 9 s pulse-off; 1 min). After centrifugation (12000 x g, 30 mins, 4°C), the supernatant was filtered (0.45 μm syringe filter, Iwaki Co., Ltd.), and loaded onto a 1 ml glutathione sepharose GSTrap FF column (GE Healthcare Life Sciences, USA) at 0.2 ml/min, with ice cooling. After washing with five column volumes of ice-cold binding buffer, proteins were eluted with 5 ml binding buffer containing 10 mM reduced glutathione (Sigma-Aldrich, USA), collecting 1.0 ml fractions. Protein fractions were analyzed on 12%, 15% or 20% SDS-PAGE gels, using colloidal Coomassie Brilliant Blue G-250 staining (Bio-Rad, USA).

Analogous procedures were used for recombinant *Z. mobilis* ATCC 29191 and CU1 Rif2 strains, except that cultures (800 ml) were grown in RM media containing 100 μg/ml Cm at 30°C to an OD_600nm_ of *ca*. 1.5-2.0. Cell cultures were incubated semi-aerobically or anaerobically (in pre-reduced RM medium) in an anaerobic chamber (Forma Anaerobic System, Thermo Fisher Scientific), using a gas mixture of 85% nitrogen, 10% carbon dioxide and 5% hydrogen; as indicated in the text. Cultures of wild type *Z. mobilis* ATCC 29191 or CU1 Rif2 were analogously used as negative controls. The mass of pelleted cells obtained from 800 ml cultures was routinely *ca*. 2.5-3 g. Respective pZ7-GST plasmid-based protein expression levels were estimated by comparing band intensities on SDS-PAGE gels with those of a dilution series of purified recombinant GST protein of known concentration. Individual protein bands were carefully excised using a sterile scalpel, and were analyzed by a combination of mass spectrometric methods: peptide mass fingerprinting (PMF) of tryptic fragments, and LC-MS/MS analysis and peptide sequencing (Proteomic Laboratory for Systems Biology Research, Baptist University of Hong Kong, Hong Kong SAR).

### Analysis of pZ7C plasmid-based GST fusion protein expression by Western Blotting

After resolution on 12% SDS-polyacrylamide gels, proteins present in the fractions eluted from GST-affinity columns were wet-transferred to polyvinylidene fluoride (PVDF) membranes using transfer buffer (25 mM Tris-HCl pH 8.3, 190 mM glycine, 20% methanol). Membranes were blocked using blocking buffer [Tris-buffered saline with Tween 20 (TBST) containing 5% non-fat milk powder] for 1 hour at room temperature. Membranes were incubated with anti-GST primary antibody (Sigma Aldrich, USA; cat. #G7781) in blocking buffer (1:2500 dilution) for 12 hours at 4°C. After washing three times with TBST, membranes were incubated with secondary antibody (HRP-linked anti-rabbit IgG; Cell Signaling Technology, USA; cat. #7074P) in blocking buffer (1:2500 dilution) for 2 hours at room temperature; before being washed three times in TBST. The membrane blots were visualized chemiluminescently using SuperSignal West Pico Chemiluminescent Substrate (Pierce Biotechnology, Thermo Scientific, USA; cat. #34079), capturing images using a Bio-Rad ChemiDoc XRS instrument (Bio-Rad, USA).

The plasmid sequences pZMO1A and pZMO7 were deposited to the NCBI GenBank database with the accession numbers **NC_019198** and **NC_019300**, respectively.

## Results

### Native plasmids in *Z. mobilis* NCIMB 11163

The NCIMB 11163 strain of *Z. mobilis* contains two relatively small native plasmids: pZMO1A (1,647 bp) and pZMO7 (4,551 bp) (Figure 1, Additional file 2). Plasmid pZMO1A has a G + C content of *ca*. 98.5% and shares 96.7% nucleotide identity (1597/1652 nt; 6 gaps) with plasmid pZMO1 (1,651 bp) from *Z. mobilis* ATCC 10988 [21,43]. As noted above, plasmid pZMO7 corresponds to plasmid p11163_3 (pZA1003), which was reported by Kouvelis *et al*. during their sequencing of the NCIMB 11163 genome [36]. Taken together, data indicates that the NCIMB 11163 strain contains four native plasmids.

### Sequence analysis of pZMO7 (pZA1003)

Plasmid pZMO7 has two predicted coding DNA sequences (CDS): pZMO7_01 (978 bp) and pZMO7_02 (1,449 bp). The pZMO7_01 CDS encodes a 326 aa replication initiation protein (Rep) **[GenBank: YP_006962143]**, which belongs to the Rep_3 superfamily (pfam01051). The pZMO7_02 CDS encodes a 483aa mobilase/replicase protein (Mob) **[GenBank: YP_006962142]**, which belongs to the relaxase/mobilisation nuclease domain family (pfam03432).

The region between the *mob* and *rep* genes on pZMO7 (positions 424 to 699) contains the predicted plasmid replication origin (*ori*). As may be seen in Figure 1 and Additional file 3, the *rep* [positions 699 (ATG) to 1679 (TAA)] and *mob* [positions 3524 (ATG) to 424 (TAA)] genes are orientated in the same direction. Putative promoter start sites predicted using a Neural Network Promoter Prediction (NNPP) programme [44] suggest that the transcription of both the *rep* and downstream *mob* genes are driven by a single promoter. Regions putatively involved in transcriptional and translational regulation are highlighted in Additional file 3.

### Construction of *E. coli* - *Z. mobilis* shuttle vectors derived from pZMO7

Previous reports have indicated that plasmids must encode both a replication origin and partnering replicase protein for stable, independent replication in *Z. mobilis* cells [23]. The *Hin*dIII/*Bam*HI fragment of pZMO7 (positions 1 to 1,876) contains the 3’-end of the *mob* gene, the predicted plasmid replication origin and the entire *rep* gene along with a *ca.* 200 bp 3’-downstream region (see Figure 1 and Additional file 3). We incorporated this ‘replicon’ fragment into two different *E. coli* plasmid backbones (pACYC-184 and pUC18), in order to determine its potential utility for shuttle vector construction. The plasmid construction strategy is outlined in Figure 2. The pZ7-184 (5,773 bp) and pZ7C (5,430 bp) plasmids contain the same 1,876 bp *Hin*dIII/*Bam*HI fragment from pZMO7, but on a pACYC-184 and pUC18 backbone, respectively.

### Qualitative evaluation of pZMO7-derived shuttle vector stability in *Z. mobilis* under selective culture conditions

To determine the potential utility of pZMO7-derived shuttle vectors for heterologous gene expression in *Z. mobilis*, we first investigated the stability of pZ7C within three different strain lineages: NCIMB 11163, ATCC 29191 (the phenotypic centrotype strain) [1], and CU1 Rif2 (which is derived from ATCC 10988) [20,45]. The pZ7C plasmid could be transformed into all three strains by electroporation, with transformation efficiencies of *ca*. 7 × 10^-6^ for the NCIMB 11163 strain, *ca*. 8 × 10^-8^ for CU1 Rif2 and *ca*. 15 × 10^-6^ for ATCC 29191 (reported as Cm-resistant colony forming units/total colony forming units surviving electroporation). Plasmid pZ7C was stably maintained for more than 150 generations in all three strains when cells were cultured in RM medium containing 100 μg/ml chloramphenicol (data not shown). An agarose gel of (*Hind*III-digested) plasmid DNA present in the three wild type (WT) and pZ7C-transformed strains is shown in Additional file 4 (Panels A, B and C: compare the lanes marked ‘WT’ and ‘pZ7C + Cm’, respectively). The introduction of pZ7C appeared to have little effect on the respective levels of the endogenous plasmids within the ATCC 29191 and CU1 Rif2 strains. However, when the recombinant NCIMB 11163/pZ7C strain was propagated in RM medium containing chloramphenicol, the intensity of the band corresponding to the endogenous pZMO7 plasmid decreased markedly compared to the wild type strain (Additional file 4, Panel A). This finding indicates that there is most probably direct competition for replication between the endogenous pZMO7 plasmid and the pZ7C shuttle vector within the same cell. However, the introduction of pZ7C had no apparent effects on the levels of the smaller endogenous pZMO1A plasmid, suggesting that it utilized a non-competing mode of replication. Equivalent results were obtained with the pZ7-184 plasmid (data not shown).

### Qualitative evaluation of pZ7C plasmid stability under non-selective culture conditions

The stability of pZ7C within the NCIMB 11163, CU1 Rif2 and ATCC 29191 strains during propagation under non-selective conditions was investigated using a previously described approach [41]. As may be seen in Additional file 4, the levels of the pZ7C plasmid remained relatively constant within the CU1 Rif2 and ATCC 29191 strains during this process of serial sub-culturing under non-selective conditions. This indicated that a selectable marker was not essentially required for stable maintenance of the pZ7C plasmid for a period of *ca*. 50-70 generations in the ATCC 29191 and CU1 Rif2 strains. The situation was markedly different in the NCIMB 11163 strain, where pZ7C levels dropped to barely detectable amounts only 24 hours (10-14 generations) after the removal of the selectable marker (Additional file 4, Panel A). This was further verified by results from quantitative PCR (qPCR) experiments performed under analogous conditions (see below).

### Copy number determination for native pZMO1A and pZMO7 plasmids in *Z. mobilis* NCIMB 11163

Before performing a more detailed analysis of their plasmid copy numbers (PCN), we first determined the relative proportions of the endogenous pZMO1A and pZMO7 (pZA1003) plasmids present within *Z. mobilis* NCIMB 11163 using a gel-based approach. The respective band intensities indicated that the PCN of pZMO1A was approximately 2-3 times that of pZMO7 (Additional file 2).

Quantitative real time PCR (qPCR) was used for a more accurate determination of the respective plasmid copy numbers, according to the method described by Skulj *et al.*[42]. Using this relative quantification approach, the PCN is determined by quantifying the number of plasmid molecules per chromosome molecules in each sample using specific qPCR primer sets. We designed two sets of qPCR primers for each plasmid, which targeted distinct loci: the *rep* and *mob* genes of pZMO7, as well as the *rep* gene and a non-coding region of the pZMO1A plasmid (see Additional file 1). The polyphosphate kinase 2 (*ppk2*) gene, a highly-conserved single copy gene present on the chromosomes of all characterized *Z. mobilis* strains [ATCC 29291: ZZ6_0566; NCIMB 11163: Za10_0556; ATCC10988 (CU1 Rif2 parent): Zmob_0569] was selected as a reference genetic locus for the determination of *Z. mobilis* chromosome copy number.

The two respective pairs of qPCR primers that targeted distinct regions on the pZMO1A or pZMO7 plasmids were then directly compared, to investigate whether or not there were notable differences in the PCN values obtained. The PCN for pZMO7 was determined to be 1.2 ± 0.1 when the *rep* gene was targeted, and was 1.4 ± 0.1 when the *mob* gene was targeted. In analogous experiments, the PCN of pZMO1A was found to be 5.0 ± 0.2 using the primer pair that targeted the *rep* gene, and was 5.3 ± 0.4 using the primer pair that targeted a predicted non-coding region of the plasmid. This data correlated closely with the estimates of relative pZMO1A and pZMO7 plasmid abundances determined using gel-densitometry (see above). The consistent nature of the PCN values obtained indicated that both of the respective pairs of qPCR primers had equivalent target specificities and amplification efficiencies.

We next used qPCR to investigate whether the PCNs of pZMO7 and pZMO1A in cultured *Z. mobilis* NCIMB 11163 cells varied considerably during the different phases of growth (Additional file 5). It was found that PCN of pZMO7 was relatively consistent throughout the growth phases, fluctuating slightly at around 1.2 copies per chromosome. The PCN of pZMO1A was around 4.5 to 5 during the lag and exponential phases, declining to around 3.0 during the stationary phase.

### Copy number determination for pZMO7-derived shuttle vectors in the *Z. mobilis* NCIMB 11163, ATCC 29191 and CU1 Rif2 strains

A similar qPCR strategy was employed to investigate the copy numbers of the pZMO7-derived pZ7C and pZ7-184 plasmids, which had been established within the *Z. mobilis* NCIMB 11163, ATCC 29191 and CU1 Rif2 strains. We designed and utilized a qPCR primer pair targeting the chloramphenicol acetyl transferase (*cat*) gene; so that the PCNs of pZ7C and pZ7-184 could be distinguished from those of the native pZMO7 plasmids within the NCIMB 11163 strain (Additional file 1). This enabled PCNs to be directly compared between the three strains. Results are summarized in Table 2. The respective PCNs determined for the pZ7C and pZ7-184 vectors were in good agreement with one another, within each of the three strains tested. These shuttle vectors were respectively maintained at *ca.* 1-2 copies per cell within the NCIMB strain, and *ca.* 2-3 copies per cell in the CU1 Rif2 strain. Copy numbers were notably higher in the ATCC 29191 strain, where the plasmids were respectively present at *ca.* 20-30 copies per cell.

**Table 2:** **Plasmid copy number determination for pZ7C and pZ7-184 in *Z. mobilis *** **NCIMB 11163, CU1 Rif2 and ATCC 29191 strains**

***Z. mobilis *** **host strain and established shuttle vector**	**Plasmid copy number**
**NCIMB 11163**	
pZ7C	1.8 ± 0.2
pZ7-184	1.2 ± 0.2
**CU1 Rif2**	
pZ7C	1.7 ± 0.3
pZ7-184	2.8 ± 0.3
**ATCC 29191**	
pZ7C	25.1 ± 1.4
pZ7-184	21.8 ± 1.6

Quantitative PCR (qPCR) was used to determine shuttle vector copy number determined using primers targeting the chloramphenicol acetyltransferase (cat) gene. Strains were cultivated in RM media containing 100 μg/ml chloramphenicol (Cm) at 30°C for 24 hours.

**Figure 3:**
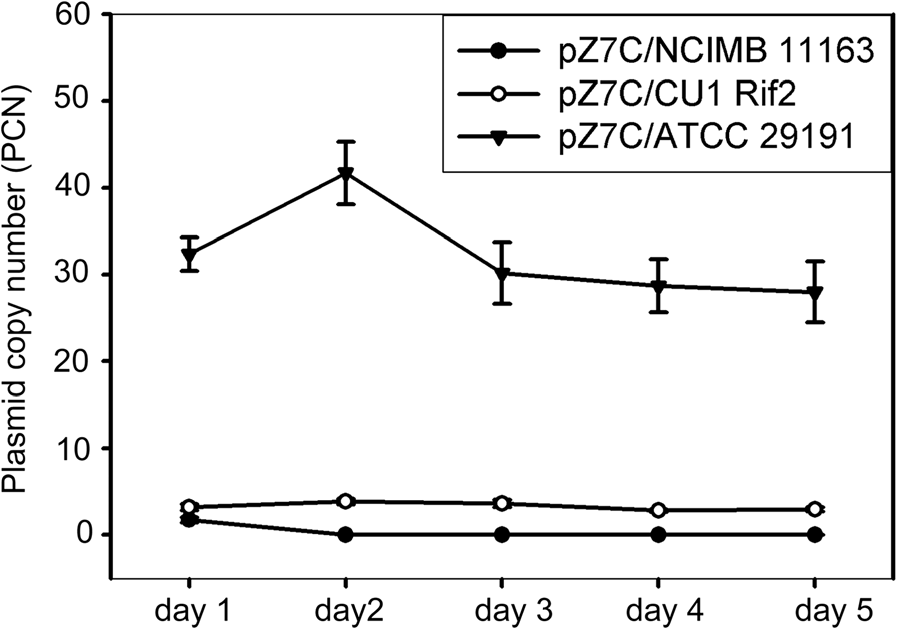
**Quantitative PCR (qPCR) analysis of pZ7C stability in *Z. mobilis *NCIMB 11163, CU1 Rif2 and ATCC 29191 strains cultured in media lacking chloramphenicol.** The plasmid copy numbers of the pZ7 shuttle vector were monitored daily using qPCR, during iterative sub-culturing of the respective recombinant strains in RM media lacking chloramphenicol. Experiments are analogous to those shown in Figure 3. See methods section for detailed experimental procedures.

Quantitative PCR was then used to evaluate pZ7C plasmid copy numbers in the ATCC 29191, CU1 Rif2 and NCIMB11163 strains during daily sub-culturing under non-selective conditions over 5 consecutive days. Results are summarized in Figure 3. In the NCIMB 11163 strain, levels of the pZ7C shuttle vector reduced to *ca*. 0.01 copies per cell, 24 hours after the removal of the chloramphenicol selectable marker (i.e. after *ca*. 10-14 generations). By the fifth day, this had fallen to *ca.* 0.002 copies per cell (i.e. *ca*. 1 plasmid molecule per 500 cells). In the CU1 Rif2 strain, the PCN for pZ7C varied from 3.8 to 2.8 over the five days. In the ATCC 29191 strain, pZ7C levels varied between 28.0 and 41.7 copies per cell. These results indicated that the PCN of the pZMO7-derived pZ7C shuttle vector remained relatively stable for at least *ca*. 50-70 cell generations in these two strains, the absence of a selectable marker. This was fully-consistent with results from the agarose gel-based analysis of pZ7C plasmid stability in these two strains.

### Construction of the pZ7-GST *Z. mobilis* expression vector

We selected the bacterial P_tac_ promoter to drive gene expression from the shuttle vector, as this approach has previously been shown to work effectively in *Z. mobilis* cells [29,46]. We designed a strategy whereby the (heterologous) gene of interest would be cloned as in-frame N-terminal fusion to the glutathione S-transferase (*gst*) gene. This would enable the straightforward detection and efficient one-step purification of any expressed GST–fusion protein within cell lysates, through the use of affinity chromatography with glutathione-derivatized resin. This approach would also enable the analysis of GST-fusion protein expression levels by Western Blotting, using anti-GST antibodies (see below). To achieve this, a DNA cassette that included the P_tac_ promoter, consensus ribosomal binding site, *gst* gene, multiple cloning site (MCS) and downstream terminator (Term) sequence (P_tac_–*gst*–MCS–Term); was inserted into pZ7C to produce pZ7-GST (Figure 2). The (heterologous) genes of interest may be cloned into the pZ7-GST expression vector via a variety of commonly-used restriction sites present in the MCS. In this plasmid, the P_tac_–*gst*–MCS–Term cassette is inserted in the opposite orientation to the P_lac_ promoter that originates from the pUC18 backbone. This ensured that transcription of the GST–heterologous gene fusions would be under the primary control of the P_tac_ promoter. As the *lacI* gene, which encodes the LacI repressor protein was not included on the pZ7-GST plasmid; gene expression would not be expected to be repressed under normal growth conditions.

### Analysis of plasmid-based Glutathione S-Transferase (GST) expression in *E. coli*, *Z. mobilis* ATCC 29191 and CU1 Rif2 strains

To determine the effectiveness of this gene-expression strategy, we first analyzed GST protein expression levels from the pZ7-GST plasmid established within *E. coli* BL21 (DE3) and *Z. mobilis* ATCC 29191 and CU1 Rif2 cells. The cell lysate proteins captured by glutathione-affinity chromatography were analyzed by SDS-PAGE (see Additional file 6, Panels A-D). It was found that the fractions eluted from the affinity-columns loaded with the *E. coli* BL21 (DE3)/pZ7-GST (Panel A), *Z. mobilis* ATCC 29191/pZ7-GST (Panel B) and CU1 Rif2/pZ7-GST (Panel C) cell lysates, all contained a band at *ca.* 26 kDa. Analysis via mass spectrometry confirmed that this band corresponded to recombinant (plasmid-derived) GST. The weak band at *ca.* 29 kDa which was apparent in the lysate prepared from wild type *Z. mobilis* ATCC 29191 (Additional file 6, Panel D), was identified as endogenous glutathione S-transferase (ZM-GST) from *Z. mobilis* ATCC 29191 (glutathione S-transferase domain protein, ZZ6_0208; 223 aa). This protein was not observable in the fractions eluted from *Z. mobilis* ATCC 29191/pZ7-GST, presumably due to its relatively low abundance compared to the recombinant GST.

The fractions eluted from the affinity-columns loaded with *Z. mobilis* ATCC 29191, ATCC 29191/pZ7-GST and CU1 Rif2/pZ7-GST cell lysates all contained a common protein band with a molecular mass of *ca.* 12 kDa (Additional file 6; Panels B, C and D), which did not appear in the purified *E. coli* fractions (Additional file 6, Panel A). This was subsequently identified as the 13.5 kDa glyoxalase/bleomycin resistance protein/dioxygenase (Glo, ZZ6_1397; 128 aa). This enzyme requires a catalytic amount of reduced glutathione, to detoxify methylglyoxal and its derivatives via S-D-lactoylglutathione to D-lactate [47], suggesting that it may contain a glutathione binding pocket. This factor may explain its notable affinity towards the glutathione-derivatized sepharose resin.

### Effects of pZ7C-GST plasmid maintenance on growth rates

We next investigated whether the presence of the pZ7C-GST expression vector significantly affected the growth rates of the NCIMB 11163, ATCC 29191 and CU1 Rif2 stains. The cell doubling times were as follows: NCIMB 11163, 104 ± 7 minutes; NCIMB 11163/pZ7-GST, 139 ± 13 minutes; CU1 Rif2, 95 ± 4 minutes; CU1 Rif2/pZ7C-GST, 111 ± 5 minutes; ATCC 29191, 85 ± 6 minutes; ATCC 29191/pZ7GST, 102 ± 9 minutes (Additional file 7). These results indicated that the maintenance of the pZ7C-GST expression vector led to only modest decreases in the growth rates (*ca.* 15-35%), compared to the respective wild type strains.

### Expression of GST-fusion proteins from pZ7C-derived vectors in *E. coli* and *Z. mobilis*

To demonstrate the applicability of the pZMO7-derived shuttle vectors for proteomic and biotechnological applications in *Z. mobilis*, we selected five proteins for expression analysis and binding-interaction analysis in the ATCC 29191 strain: acyl-carrier protein (AcpP, ZZ6_0066; 78 aa), 2-dehydro-3-deoxyphosphooctonate aldolase (KdsA, ZZ6_1604; 292 aa), chaperone protein DnaJ (ZZ6_0618; 375 aa), RNA chaperone Hfq (ZZ6_0899; 161 aa) and DNA polymerase III chi subunit (HolC, ZZ6_0042; 148 aa). These proteins were previously included in a large scale analysis of protein-protein binding interactions in *E. coli*[35]. All five genes were successfully cloned into the pZ7-GST expression vector, creating the respective N-terminal GST fusions: pZ7-GST-acpP; pZ7-GST-kdsA; pZ7-GST-dnaJ; pZ7-GST-hfq and pZ7-GST-holC. We first qualitatively determined the respective expression levels of the five pZ7-GST plasmid-encoded GST-fusion proteins within *E. coli* BL21 (DE3); including plasmid pZ7-GST as a positive control. SDS-PAGE gels of the cell lysate proteins eluted from the GST-affinity columns are shown in Additional file 8. It was found that the recombinant GST, GST-AcpP, GST-Hfq and GST-KdsA proteins were expressed to detectable levels; with levels of GST-AcpP being the highest. Plasmid-encoded GST-fusions of the DnaJ and HolC proteins were not expressed to visually detectable levels.

**Figure 4:**
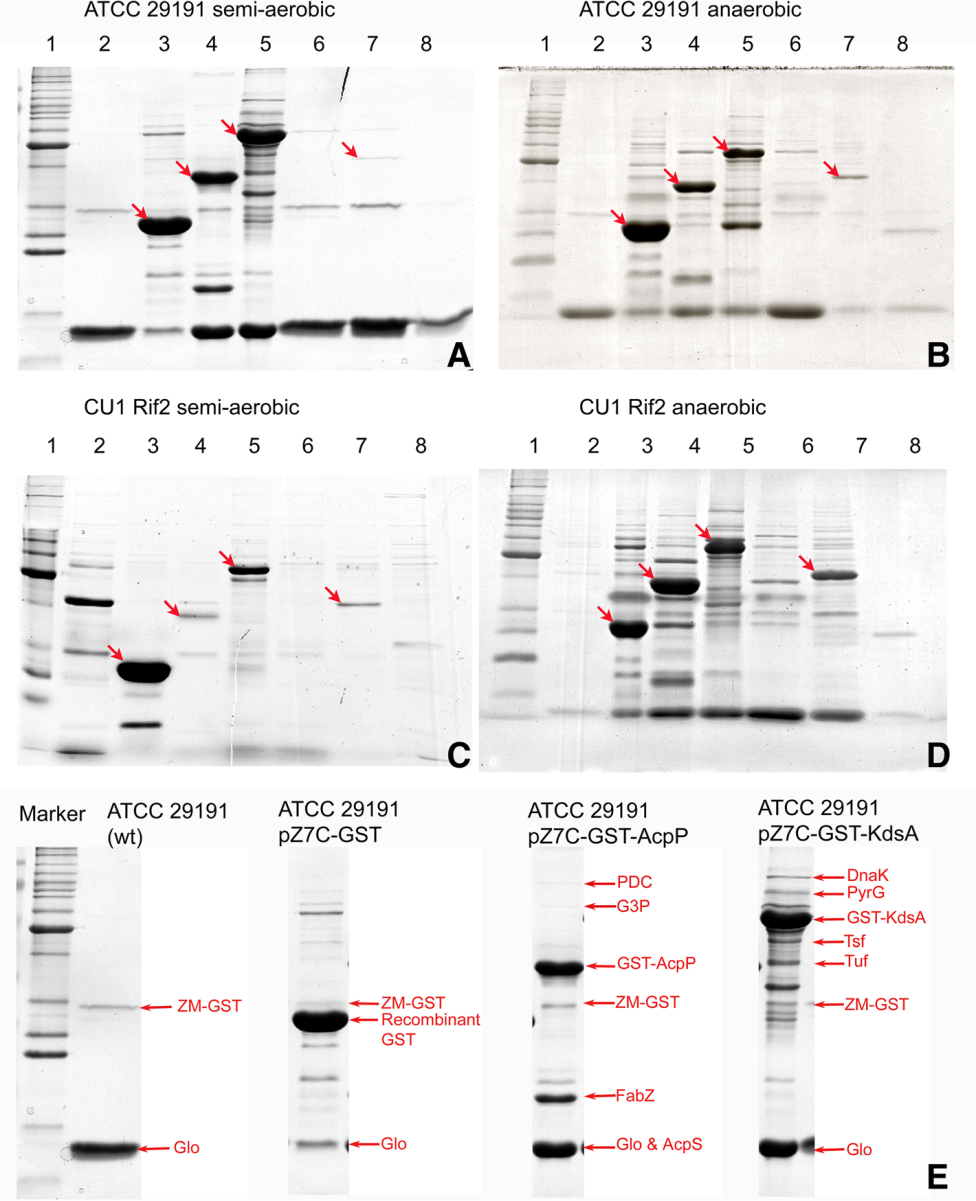
**Analysis of pZ7-GST-fusion protein expression patterns and affinity-purified protein complexes in *Z. mobilis.*** 15% SDS-polyacrylamide gels (Coomassie Blue-stained) of proteins obtained after glutathione-affinity chromatography of cell lysates prepared from cultures of wild-type or transformant strains of *Z. mobilis* containing pZ7-GST, or pZ7-GST-derived expression vectors. **Panel A**: *Z. mobilis* ATCC 29191 wild type and plasmid transformed strains grown under semi-aerobic conditions. **Panel B**: *Z. mobilis* ATCC 29191 wild type and plasmid transformed strains grown under anaerobic conditions. **Panel C**: *Z. mobilis* CU1 Rif2 wild type and plasmid transformed strains grown under semi-aerobic conditions. **Panel D**: *Z. mobilis* CU1 Rif2 wild type and plasmid transformed strains grown under anaerobic conditions. The eluted protein fractions shown in lanes 1-8 are equivalent in Panels A-D. Red arrows indicate the positions of the respective pZ7C-GST-fusion proteins. **Lane 1**: Benchmark protein ladder; **lane 2**: wild type *Z. mobilis* strain (no shuttle vector); **lane 3**: pZ7-GST; **lane 4**: pZ7-GST-AcpP; **lane 5**: pZ7-GST-KdsA; **lane 6**: pZ7-GST-DnaJ; **lane 7**: pZ7-GST-Hfq; **lane 8**: pZ7-GST-HolC. **Panel E**: From left to right, identities of the proteins (co-purifying complexes) obtained from lysates of wild type (wt) *Z. mobilis* ATCC 29191; *Z. mobilis* ATCC 29191/pZ7C-GST; *Z. mobilis* ATCC 29191/pZ7C-GST-AcpP; and *Z. mobilis* ATCC 29191/pZ7C-GST-KdsA; grown under semi-aerobic conditions. ZM-GST: native glutathione S-transferase domain protein (ZZ6_0208); Glo: glyoxalase/bleomycin resistance protein/dioxygenase (ZZ6_1397); Recombinant GST: heterologous recombinant GST expressed from pZ7-GST; GST-AcpP: recombinant GST-AcpP fusion protein; GST-KdsA: recombinant GST-KdsA fusion protein; PDC: pyruvate decarboxylase (ZZ6_1397); AcpS: holo-acyl-carrier-protein synthase (ZZ6_1409); PyrG: CTP synthase (ZZ6_1034); DnaK: chaperone protein DnaK (ZZ6_0619); Tsf: translation elongation factor Ts (ZZ6_0173); Tuf: translation elongation factor Tu (ZZ6_0750); FabZ: (3R)-hydroxymyristoyl-ACP dehydratase (ZZ6_0182); G3P: glyceraldehyde-3-phosphate dehydrogenase (ZZ6_1034).

Analogous protein expression experiments were then performed in the ATCC 29191 and CU1 Rif2 strains of *Z. mobilis*. To investigate whether there were significant differences in plasmid-based protein expression patterns during different metabolic/respiratory modes of growth, the respective wild type and transformed strains were cultured under both semi-aerobic and anaerobic conditions. SDS-polyacrylamide gels of the respective eluted fractions are shown in Figure 4, Panels A-D. The GST, GST-AcpP, GST-KdsA and GST-Hfq proteins were expressed to visually-detectable levels in both the ATCC 29191 and CU1 Rif2 strains, under both conditions (indicated with red arrows in Figure 4). It may be seen that there were only minor inter-strain differences in the relative expression levels of the plasmid-encoded proteins under semi-aerobic or anaerobic conditions.

Western blotting experiments using anti-GST antibodies were performed to confirm the identities of the recombinant GST-fusion proteins observed on the SDS-polyacrylamide gels. This technique also enabled the detection of GST-containing proteins present at low levels, as well as ones that had been otherwise modified within the cell. The gel blots of the plasmid-encoded GST and 5 GST-fusion proteins respectively expressed in the ATCC 29191 and CU1 Rif2 strains are shown in Additional file 9. Consistent with the Coomassie stained gel images, the expression of the GST, GST-AcpP, GST-KdsA and GST-Hfq proteins was clearly apparent (indicated with red arrows in Figure 4 and Additional file 9). However, the blots also revealed that the GST-DnaJ protein was also expressed in both strains; with a partially-degraded form predominating in the CU1 Rif2 strain (apparent molecular weight of *ca*. 55-58 kDa, compared to the predicted 67.7 kDa for the full length GST-fusion).

To further probe the utility of pZ7C-derived shuttle vectors for biotechnological applications in *Z. mobilis*, we quantified the respective levels of recombinant GST and GST-fusion proteins expressed from the pZ7-GST, pZ7-GST-acpP and pZ7-GST-kdsA vectors established in the ATCC 29191 strain, when cultured under semi-aerobic conditions to an OD_600nm_ of *ca*. 1.5-2. Results indicated that *ca*. 5 mg of recombinant GST, 2-3 mg of GST-AcpP and 4 mg of GST-KdsA were expressed and recovered from 2.5-3 g wet cell mass of the respective *Z. mobilis* ATCC 29191 transformant strains.

### *Z. mobilis* protein binding interaction analysis via GST-affinity chromatography

Bands were carefully excised from the SDS-PAGE gels of fractions eluted from the GST-affinity columns, so that co-purifying protein species and/or background proteins could be identified via mass spectrometry. As may be seen in Figure 4, the *ca*. 12 kDa glyoxalase/bleomycin resistance protein (Glo) and *ca*. 29 kDa glutathione-S-transferase (ZM-GST) were commonly observed in eluted fraction from the plasmid-free control and all transformant strains. Even with the propitious use of protease inhibitors, a complex, heterogeneous mixture of low molecular weight proteins/protein fragments co-migrated with the Glo protein, near the gel front. Proteins that were respectively co-purified with either the GST-AcpP or GST-KdsA ‘bait’ proteins, but were absent in all other eluted fractions, were identified as forming putative binding interactions (Table 3). The four identified protein species that co-purified with recombinant GST-AcpP were: pyruvate decarboxylase (PDC; ZZ6_1712), glyceraldehyde-3-phosphate dehydrogenase (G3P; ZZ6_1034), (3R)-hydroxymyristoyl-ACP dehydratase (FabZ; ZZ6_0182) and holo-acyl-carrier-protein synthase (AcpS; ZZ6_1409). The four identified protein species that co-purified with GST-KdsA were: translation elongation factor Ts (Tsf; ZZ6_0173); translation elongation factor Tu (Tuf; ZZ6_0750); cytidine 5’-triphosphate (CTP) synthase (PyrG; ZZ6_0800) and chaperone protein DnaK (ZZ6_0619). None of these proteins were identified in controls. It may be noted that not all of the (unique) co-purifying proteins could be unambiguously identified.

**Table 3:** **Identities of proteins purified by glutathione-affinity purification of cell lysates prepared from cultured wild type and transformant *Z. mobilis *** **ATCC 29191 strains**

**Expression vector used**	***Z. mobilis *** **proteins identified**	**Corresponding locus tag (gene)**
-	Glyoxalase/bleomycin resistance protein/dioxygenase Glo	ZZ6_1397 (*glo*)
	Glutathione S-transferase domain protein	ZZ6_0208
pZ7-GST	Glyoxalase/bleomycin resistance protein/dioxygenase Glo	ZZ6_1397 (*glo*)
	Recombinant GST (from expression vector)	-
	Glutathione S-transferase domain protein	ZZ6_0208
pZ7-GST-acpP	Glyoxalase/bleomycin resistance protein/dioxygenase Glo	ZZ6_1397 (*glo*)
	Holo-acyl-carrier-protein synthase AcpS	ZZ6_1409 (*acpS*)
	(3R)-hydroxymyristoyl-ACP dehydratase FabZ	ZZ6_0182 (*fabZ*)
	Glutathione S-transferase domain protein	ZZ6_0208
	Acyl carrier protein AcpP	ZZ6_0066 (*acpP*)
	Glyceraldehyde-3-phosphate dehydrogenase G3P	ZZ6_1034 (*gap*)
	Pyruvate decarboxylase PDC	ZZ6_1712 (*pdc*)
pZ7-GST-kdsA	Glyoxalase/bleomycin resistance protein/dioxygenase Glo	ZZ6_1397 (*glo*)
	Glutathione S-transferase domain protein	ZZ6_0208
	2-dehydro-3-deoxyphosphooctonate aldolase KdsA	ZZ6_1604 (*kdsA*)
	Translation elongation factor Ts	ZZ6_0173 (*tsf*)
	Translation elongation factor Tu	ZZ6_0750 (*tuf*)
	CTP synthase PyrG	ZZ6_0800 (*pyrG*)
	Chaperone protein DnaK	ZZ6_0619 (*dnaK*)

## Discussion

Even though the NCIMB 11163 (formerly NCIB 11163, also known as B70) strain of *Z. mobilis* was included in the seminal review by Swings and De Ley [1], and its genome sequence was announced in 2009 [36], it has been the subject of very few investigations in the scientific literature. Consequently, there are no reports concerning the properties of its native plasmids, nor reports of their use for the construction of recombinant plasmids. Here, we describe the construction, intracellular stability and biological utilization of shuttle-vectors derived from the pZMO7 (pZA1003) native plasmid from this strain.

### Replication and stability of pZMO7-derived recombinant plasmids in *Z. mobilis*

The pZMO7-derived shuttle vectors constructed here (pZ7-184, pZ7C and its derivatives) could be transformed into three distinct lineages of *Z. mobilis,* without apparent genetic (structural) alteration upon prolonged culture or repeated subculture. Our data suggests that there is direct competition between the native pZMO7 plasmids and recombinant pZMO7-derived shuttle vectors for access to the replication-related machinery within the NCIMB 11163 strain. This hypothesis is consistent with the previously-proposed competition between native pZMO2 and recombinant pZMO2-derived plasmids (e.g. pDS212) in the CU1 Rif2 strain [20]. However, this competition for plasmid replication appears to be largely absent in the ATCC 29191 and CU1 Rif2 strains. Homologues of the Rep_3 superfamily replicase protein [**pfam01051**] encoded by pZMO7 do not appear to be present in plasmids belonging to any *Z. mobilis* strain sequenced to date, including ATCC 29191, ATCC 31821 and ATCC 10988. These results suggest that the pZMO7-derived Rep_3 superfamily replicase protein and/or its corresponding plasmid replication origin function orthogonally to other *Z. mobilis* plasmid replication systems. The cellular stabilities and replicative properties of pZMO7-derived shuttle vectors within other *Z. mobilis* lineages remains to be established.

The copy numbers of the pZ7C and pZ7-184 shuttle vectors varied considerably in the three *Z. mobilis* strains tested. However, their respective PCNs were closely matched within the same strain (Table 2). This indicated that the respective pUC18 or pACYC-184 derived plasmid backbones had little effect on their replication properties within *Z. mobilis*; which were primarily governed by the *ca.* 1,900 bp replicon fragment from pZMO7. Copy number was highest in the ATCC 29191 strain (*ca.* 20-40 plasmids per cell), and considerably lower in the NCIMB 11163 and CU1 Rif2 strains (*ca.* 1-3 plasmids per cell). Further detailed studies will be required to establish the physiological basis for this inter-strain variation in PCN.

### Protein expression and proteomic applications within *Z. mobilis*

To demonstrate a proof of principle, we selected a well-established glutathione/glutathione S-transferase (GST) affinity ‘pull-down’ approach [34] for use in *Z. mobilis*. In addition to functioning as a convenient method for one-step protein isolation, (N-terminal) GST fusions have previously been shown to be beneficial for the expression of soluble (heterologous) proteins in bacteria [48]. The AcpP, KdsA, DnaJ, Hfq and HolC proteins selected as ‘bait’ were included in a previous proteomic study conducted in *E. coli*[35]. With the exception of the Hfq RNA chaperone [49], the respective properties of these proteins have not previously been analyzed in *Z. mobilis*. Four out of five proteins were expressed in a soluble form in both the ATCC 29191 and CU1 Rif2 strains, clearly demonstrating the effectiveness of the pZ7-GST vector-based system. The GST-HolC protein may have been expressed in an insoluble form, thus failing to be recovered in the (soluble) cell lysate fractions. Co-purifying proteins were identified for two of the four GST-fusion proteins that were expressed in the ATCC 29191 strain (AcpP and KdsA). However, it should be noted that the plasmid-based GST-fusion protein expression is performed in a wild-type chromosomal background. Consequently, the GST-tagged bait proteins will be in direct competition with the corresponding endogenous bait proteins, for the capture of binding partners within the cell. Hence it may not be possible to capture and purify sufficient levels of interacting protein species to enable their subsequent detection or identification.

The acyl carrier protein AcpP, which acts as a covalent carrier of fatty acid intermediates during their biosynthesis, co-purified with four other functionally-related enzymes. These were the pyruvate decarboxylase (PDC), which catalyzes the decarboxylation of pyruvate, an intermediate in the fatty acid cycle glyceraldehyde-3-phosphate dehydrogenase (G3P), which catalyzes the dehydrogenation of glyceraldehyde-3-phosphate, another intermediate in this cycle hydroxymyristoyl-ACP dehydratase (FabZ), which catalyzes the dehydration of short chain beta-hydroxyacyl-ACPs and long chain saturated and unsaturated beta-hydroxyacyl-ACPs); as well as and holo-acyl carrier protein synthase (AcpS) proteins [50], which is involved in the transfer of phosphopantetheine to ACP. To date, only the FabZ-AcpP and AcpS-AcpP protein binding associations have been described in the Database of Interacting Proteins (DIP) [51], STRING [52], or the Prolinks databases [53]. However, it should also be noted that we did not detect additional protein interactions that were previously observed in *E. coli*[35]; for example, 3-oxoacyl-(acyl-carrier-protein) synthase 2 (FabF), 3-oxoacyl-(acyl-carrier-protein) synthase III (FabH), malonyl CoA-acyl carrier protein transacylase (FabD) short-chain dehydrogenase/reductase SDR (FabI) were not co-purified with AcpP. This may be due to their relatively low cellular abundance under the culture conditions employed, or may be due to the fact that only relatively high-affinity or long-lasting protein-protein interactions are detected using our approach.

KdsA is involved in the early stages of lipopolysaccharide biosynthesis catalyzing the synthesis of 2-dehydro-3-deoxy-D-octonate 8-phosphate [54]. This protein was found to interact with CTP synthase (PyrG); chaperone protein DnaK; elongation factor Ts (Tsf) and elongation factor Tu (Tuf). CTP synthase plays a key role in pyrimidine biosynthesis; inter-converting the UTP and CTP nucleotides [55]. The DnaK chaperone protein is induced in response to cellular stresses such as hyperosmotic shock, and plays important roles in the replication of chromosomal and phage DNA [56]. Elongation factors Ts and Tu work together, modulating the translation of proteins at the ribosome [57]. Only the interaction between CTP synthase and KdsA is included in the current versions of the above protein-protein interaction prediction databases. It is conceivable that the other putative protein interactions may be due to functional interplay between DNA replication, translation and lipopolysaccharide biosynthesis within *Z. mobilis*. However, additional analyses, e.g. reciprocal protein binding interaction experiments are required to verify this speculation.

There have been relatively few literature reports analyzing protein expression patterns in *Z. mobilis*. More than 20 years ago, Mejia *et al.* and An *et al.* used two-dimensional gel electrophoresis to survey the proteome of *Z. mobilis* CP4 under various growth conditions, identifying *ca*. 10-20 protein spots [58,59]. Most notably, Yang *et al.* have recently conducted a comprehensive ‘systems biology’ analysis of response pathways to ethanol stress in the *Z. mobilis* ZM4 strain [60]. They used a ‘shotgun’ MudPIT proteomic approach to quantify protein expression levels under physiological conditions pertinent to ethanol production. Networks of functionally-associated proteins were defined using a combination transcriptional, proteomic and data-mining approaches. Of relevance to this study, they noted that the DnaJ chaperone protein (ZMO1690) and Hfq RNA chaperone (ZMO0347) were both up-regulated in response to stress, whilst levels of the KdsA (ZMO1488), AcpP (ZMO1279) were unchanged. Expression of the HolC DNA polymerase III chi subunit (ZMO1308) was not detected. A targeted protein-affinity ‘pull-down’ approach such as the one described here may be used to complement such large scale studies, verifying protein associations inferred by other *in silico* or experimental approaches.

## Conclusions

Whilst quantitative (real time) PCR approaches have previously been used to establish plasmid copy numbers in microbes, this is the first time it has been used to evaluate plasmid levels in *Zymomonas*, or closely-related alphaproteobacterial species. Our results indicate that shuttle vectors containing the replicon from the pZMO7 (pZA1003) native plasmid from *Z. mobilis* NCIMB 11163 may be stably maintained in multi-copy levels for more than 50 generations in the ATCC 29191 and (ATCC 10988-derived) CU1 Rif2 strains, in the absence of a selectable marker. A selectable marker is required for stable pZMO7-derived shuttle vector maintenance in the parental NCIMB 11163 strain, most probably due to replicative competition with endogenous pZMO7 plasmids. The replication of pZMO7 and pZMO1 plasmids appears to function in an orthologous, and non-competitive manner.

The pZMO7 shuttle vector-based expression of N-terminal GST-fusions of ‘bait’ proteins enables the composition of intracellular protein complexes in *Z. mobilis* to be probed in a convenient and straightforward manner. The co-purification of established and putative functionally-related proteins validates the use of this experimental approach. Taken together, our data suggests that the composition of protein complexes within *Z. mobilis* cells may share significant similarity to those found in *E. coli*, *Saccharomyces cerevisae* and other microbial species [32-35].

## Abbreviations

Amp: Ampicillin; bp: Base pair; CDS: Coding DNA sequence; Cm: Chloramphenicol; dsDNA: Double stranded deoxyribonucleic acid; E. coli: *Escherichia coli*; EDTA: Ethylenediaminetetraacetic acid; EtBr: Ethidium bromide; GST: Glutathione S-transferase; LB: Luria Broth; MCS: Multiple cloning site; nt: Nucleotide; OD: Optical density; PCN: Plasmid copy number; PCR: Polymerase chain reaction; PMF: Peptide mass fingerprinting; qPCR: Quantitative polymerase chain reaction; RM: Rich Medium; SDS-PAGE: Sodium dodecyl sulfate-polyacrylamide gel electrophoresis; Tc: Tetracycline; Z. mobilis: *Zymomonas mobilis*.

## Competing interests

The authors declare no competing interests; financial or otherwise.

## Authors’ contributions

Conceived the study: RMW, MS. Designed and performed the practical experimental work: RMW, LYS, WYC. Analyzed results and data: RMW, LYS, DCLP, WYC. Wrote the manuscript: RMW, LYS, DCLP, MS, WYC. All authors read and approved the final manuscript.

## Supplementary Material

Additional file 1 Primers used in this study. Click here for file

Additional file 2 **Restriction analysis of native plasmid DNA extracted from *Z. mobilis *** **NCIMB 11163.** Click here for file

Additional file 3 Predicted positions of open reading frames and putative gene regulatory elements on plasmid pZMO7. Click here for file

Additional file 4 **Stability of pZ7C shuttle vector in *Z. mobilis *** **NCIMB 11163, CU1 Rif2 and ATCC 29191 strains cultured in media with/without selection agent.** Click here for file

Additional file 5 **Quantitative-PCR determination of plasmid copy number for pZMO1A and pZMO7 in *Z. mobilis *** **NCIMB 11163 throughout the growth cycle.** Click here for file

Additional file 6 **Affinity-purification of recombinant GST protein expressed from plasmid pZ7-GST established in *E. coli *** **BL21 (DE3), and *Z. mobilis *** **ATCC 29191 and CU1 Rif2.** Click here for file

Additional file 7 **Growth curves for wild type and pZ7C-GST plasmid-transformed *Z. mobilis *** **strains NCIMB 11163, CU1 Rif2 and ATCC 29191.** Click here for file

Additional file 8 **Expression of GST-fusion proteins from respective pZ7-GST plasmid constructs established in *E. coli.***
Click here for file

Additional file 9 **Western blot analysis of pZ7C-GST fusion protein expression levels in *Z. mobilis *** **ATCC 29191 and CU1 Rif2.** Click here for file
