# Supplementary material for: pZMO7-Derived shuttle vectors for heterologous protein expression and proteomic applications in the ethanol-producing bacterium Zymomonas mobilis
Source: BMC Microbiol. 2014 Mar 15;14:68. doi: 10.1186/1471-2180-14-68 (PMC4004385; doi:10.1186/1471-2180-14-68)
Supplement: Additional file 1 — Primers used in this study. [file 1471-2180-14-68-S1.pdf]

Additional File 1. Primers used in this study

Primer	Sequence (5' to 3')	Application
11163F1	CAACTTAGAGCTGTTGTTATGG	Sequencing of pZMO7 plasmid
11163R1	CAGCGAAAGAAAGAACTCC	
11163F2	AAGACTTTACCTCTAGCCGAAC	
11163R2	CGCTCGACCAAGATTTCTAC	
11163F3	GAACGAACAGGAAGACATAC	
11163R3	TGTGATACCTGCCTTACTG	
Zm0716 ppk2-F	ATATGGATCCATGTCTGTCAATCTTGCTGATTACG	PCR amplification of *ppk2*
Zmp716 ppk2-R	AATTCTCGAGTTAAAGCTTTTCGCCCAGATCAATATCAAAT	
Cm-F	TTATGATATCGGCAGCATCACCCGACGCACT	PCR amplification of Cm^r gene cassette from pLysS
Cm-R	GCGCGATATCTAAGGGCACCAATAACTGCCT	
Ptac-F	ATATAGATCTTGAAATGAGCTGTTGACAATTAATCATCGGCTCG	PCR amplification of P_{tac}-*gst*-T_{tac} cassette from pGEX4T1
Ttac-R	ATATCATATGAGATCTAAACGCGCGAGGCAGATCG	
acpP-F	ATATGGATCCATGAGCGATACTGCAGAGCGCATC	PCR amplification of *acpP*
acpP-R	AATTCTCGAGTTAAGCCTTGTGCTCGTCGATATAGCTGAC	
dnaJ-F	ATATGGATCCATGGTGTCTGAGTTAGATTATTATAGTTTG	PCR amplification of *dnaJ*
dnaJ-R	AATTCTCGAGTTATTCGCCTGTTAAAATTGTTT	
hfq-F	ATATGGATCCATGGCCGAAAAGGTCAACAATC	PCR amplification of *hfq*
hfq-R	AATTCTCGAGTCAATCCTCGTCTCGCCTTTC	
holC-F	ATATGGATCCATGTTGGTAGCACGGGTTGATTTTTATCA	PCR amplification of *holC*
holC-R	AATTCTCGAGTTAGGCTATGGCCTTCCACTG	
kdsA-F	ATATGGATCCATGTCTAACAAACTAAATAACCAATATCCGAG	PCR amplification of *kdsA*
kdsA-R	AATTCTCGAGTTATTGATCAATCAAGTGGCGTTCTTTC	
Zm-ppk2-Frt	CATTTCCGGGCATTTTTGTC	Determination of *ppk2* gene (chromosomal) copy number
Zm-ppk2-Rrt	CGACTGAAGAAGAAAAAGCACATC	
pZMO7-rep-Frt	TAGCCGAGCTTAGAGGAAAACTG	Determination of copy numbers of endogenous pZMO7 and pZMO7-derived shuttle vectors by targeting at *rep* gene of pZMO7
pZMO7-rep-Rrt	GCAACATTGATCGCTTTATCTCTTAA	
continued		
pZMO7-mob-Frt	GACCGTCGAACAGCTCAACA	Determination of copy number

pZMO7-mob-Rrt	CTCCGCAATGGCTGAATTG	of pZMO7by targeting at *mob* gene of pZMO7
pZMO1A-rep-Frt	CGGAAGTAACGCGAGGAAAA	Determination of the copy number of endogenous pZMO1A buy targeting at *rep* gene of pZMO1A
pZMO1A-rep-Rrt	CCTAACCATCAATAAGCAATGAAAATG	
pZMO1A- Frt	AGCGGCTTGGCCTTGAC	Determination of the copy number of endogenous pZMO1A by targeting at non-coding region in pZMO1A
pZMO1A- Rrt	TCCTTTCCAGCACTCCAGTGA	
Cm-Frt	CCGCCCTGCCACTCATC	Determination of copy numbers of shuttle vectors (containing Cm^r cassette) by targeting at Cm^r cassette
Cm-Rrt	CCATGTCGGCAGAATGCTT	
