# Supplementary material for: pZMO7-Derived shuttle vectors for heterologous protein expression and proteomic applications in the ethanol-producing bacterium Zymomonas mobilis
Source: BMC Microbiol. 2014 Mar 15;14:68. doi: 10.1186/1471-2180-14-68 (PMC4004385; doi:10.1186/1471-2180-14-68)
Supplement: Additional file 2 — Restriction analysis of native plasmid DNA extracted from Z. mobilis NCIMB 11163. [file 1471-2180-14-68-S2.pdf]

Additional File 2

Restriction analysis of native plasmid DNA extracted from *Z. mobilis* NCIMB 11163

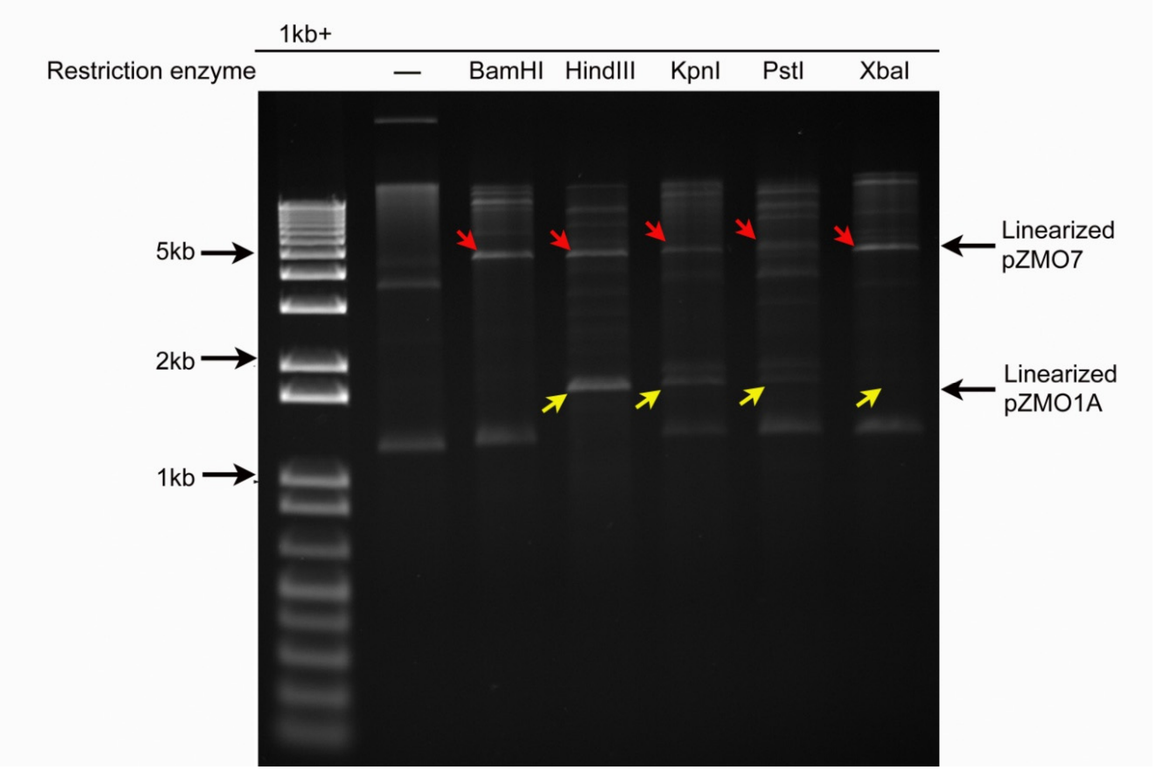

Native plasmid DNA was extracted from wild type *Zymomonas mobilis* NCIMB 11163, and aliquots were digested using the restriction enzymes indicated in the figure. Digestion products were analyzed on a 1% TAE-agarose gel using ethidium bride staining. The position of the DNA fragment corresponding to linearized pZMO7 is indicated with red arrows, and linearized pZMO1A is indicated with yellow arrows. **Lane 1**, 1kb+ DNA ladder (Invitrogen); **lane 2**, undigested plasmid DNA extracted from *Z. mobilis* NCIMB 11163; **lane 3**, plasmid DNA digested by BamHI; **lane 4**, plasmid DNA digested by HindIII; **lane 5**, plasmid DNA digested by KpnI; **lane 6**, plasmid DNA digested by PstI; **lane 3**, plasmid DNA digested by XbaI;
