# Supplementary material for: pZMO7-Derived shuttle vectors for heterologous protein expression and proteomic applications in the ethanol-producing bacterium Zymomonas mobilis
Source: BMC Microbiol. 2014 Mar 15;14:68. doi: 10.1186/1471-2180-14-68 (PMC4004385; doi:10.1186/1471-2180-14-68)
Supplement: Additional file 3 — Predicted positions of open reading frames and putative gene regulatory elements on plasmid pZMO7. [file 1471-2180-14-68-S3.pdf]

Additional File 3

Predicted positions of open reading frames and putative gene regulatory elements on plasmid pZMO7

```
        HindIII     Mob Continues
   1   AAGCTTTGCAGGAGGAAAAAGC......AGAGGTCGTTCTCGCTCTCGATAA              424
        SerPheAlaGlyGlyLysSer......ArgGlyArgSerArgSerArg*

 425   ACTTGA AAAGGACGCT AATTCCGAAA AGTTTTTTAT CTTTCTGATA GTCGATTTTA        480

                                                Predicted Term
 481   GTTGGCGATT TCCGATAAAA AAGCCCTGAA CTTATTGAAC TTTCCTATAT TTCAATAAGT    540

 541   TCAATAAGTT CAGGGCTTTT TTATTCAAAA AAATCAATGC CTTACGACTT TAAAAACCAC    600

                                          -35
 601   TTTTGGGGAG TGAAAAACCA CTTTTGGGGA GTGAAAAACC ACTTTTGGGG AGTGAAAAAC    660

             -10        TSS         RBS      Rep
 661   CACTTCAAAA AATATATTAA GTGGTTAATA AAGTTGTAATGAGAAAAACACTACAAATAA...   720
                                          MetGluTyrArgAlaLysAsp ...

1659 .. .AGAATAGAATTTAATAATTAAA ATATAATTCT ATTATTCATA GAAAGAGTTC...        1710
     . ..ArgIleGluPheAsnAsn*

                RBS     Mob
3511 .. .AGATATCAGA ATTATGATTATCGGCTTCTCAAAA...CAAGCTCTATACCCACCAGATCA    4551
                       MeTIleIleGlyPheSerLyG...GlnAlALeuTyrProProAspGln
```

Regions on plasmid pZMO7 predicted to be involved in regulating transcription and translation of the encoded *mob* and *rep* genes. The adenosine residue at the 5'-end of the *Hin*dIII site is designated position 1 for the pZMO7 plasmid. In coding regions, corresponding amino acid sequences are shown under the DNA sequence using standard 3-letter symbols. Start codons (ATG) and stop codons (TAA) are bold and underlined. Predicted promoter elements (-35, CCACTT; -10, AAAAAT), transcription start site (TSS) and ribosome-binding sites (RBS) (AAG, AGA) and transcription terminators (palindromic sequences that form stem-loop structures) are underlined and labeled above the nucleotide sequence as -35, -10, RBS, TSS and Predicted Term, respectively. The nucleotides highlighted in blue correspond to the promoter regions, as predicted by Neural network promoter prediction. A single asterisk indicates the end of the protein sequence.
