# Supplementary material for: pZMO7-Derived shuttle vectors for heterologous protein expression and proteomic applications in the ethanol-producing bacterium Zymomonas mobilis
Source: BMC Microbiol. 2014 Mar 15;14:68. doi: 10.1186/1471-2180-14-68 (PMC4004385; doi:10.1186/1471-2180-14-68)

Additional File 4

Stability of pZ7C shuttle vector in *Z. mobilis* NCIMB 11163, CU1 Rif2 and ATCC 29191 strains cultured in media with/without selection agent

Shuttle vector levels were monitored during iterative sub-culturing in RM media lacking chloramphenicol, over a period of *ca*. 50-70 generations. *Z. mobilis* NCIMB 11163, CU1 Rif2 and ATCC 29191 strains that had been freshly-transformed with pZ7C were cultured semi-aerobically in RM medium containing 100 μg/ml chloramphenicol (+Cm) for *ca*. 24 hours, to allow the shuttle vector to become established (+pZ7C, +Cm). Aliquots were expanded 1:100 into fresh RM media lacking chloramphenicol (-Cm) and were cultured semi-aerobically at 30°C for 24 hours. This sub-culturing process was repeated every 24 hours for a total of 5 days. Plasmid DNA was extracted daily, and was *Hind*III-digested (to linearize pZ7C, pZMO7 and pZMO1A plasmids), prior to analysis on Et-Br stained 1% agarose-TAE gels.

Lanes are equivalent in each panel (from left to right). **Lane 1**: 1kb+ DNA ladder; **lane 2**: Plasmid DNA extracted from wild type strains cultured in RM medium; **lane 3**: pZ7-transformed strains after 24 hours culture in RM + Cm; **lane 4**: pZ7-transformed strains after 24 hours culture in RM – Cm; **lane 5**: pZ7-transformed strains after first sub-culturing step; **lane 6**: after second sub-culturing iteration; **lane 7**: after fourth sub-culturing iteration; **lane 8**: after fifth sub-culturing iteration.

Panel A: wild type NCIMB 11163 and NCIMB 11163/pZ7C strains

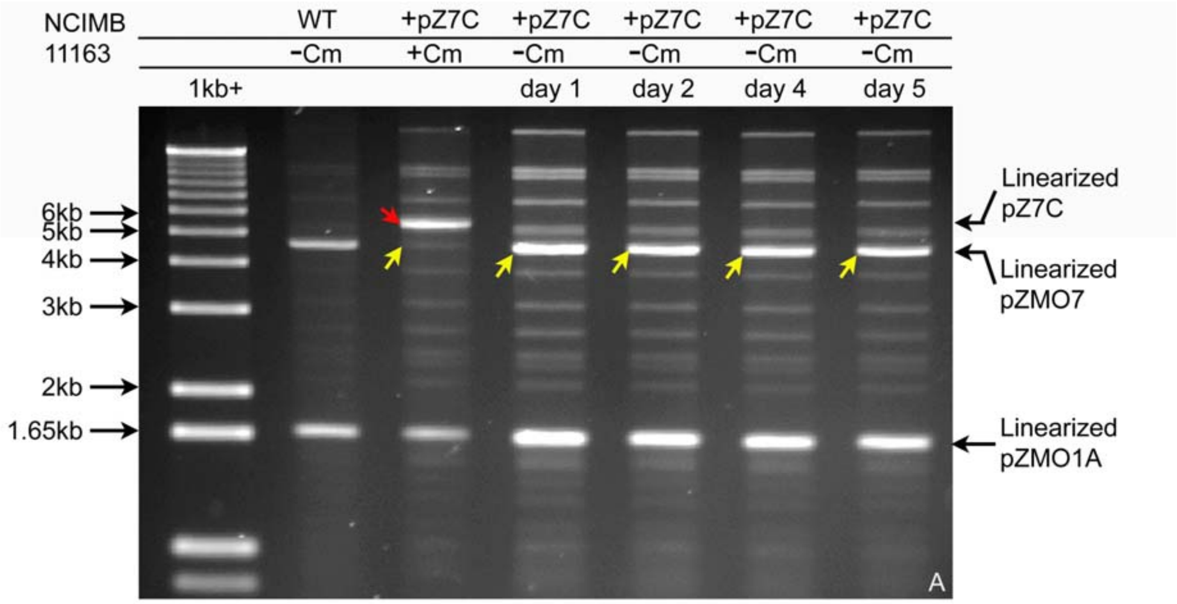

Additional File 4

Panel B: wild type CU1 Rif2 and CU1 Rif2/pZ7C strains

Panel C. wild type ATCC 29191 and ATCC 29191/pZ7C strains

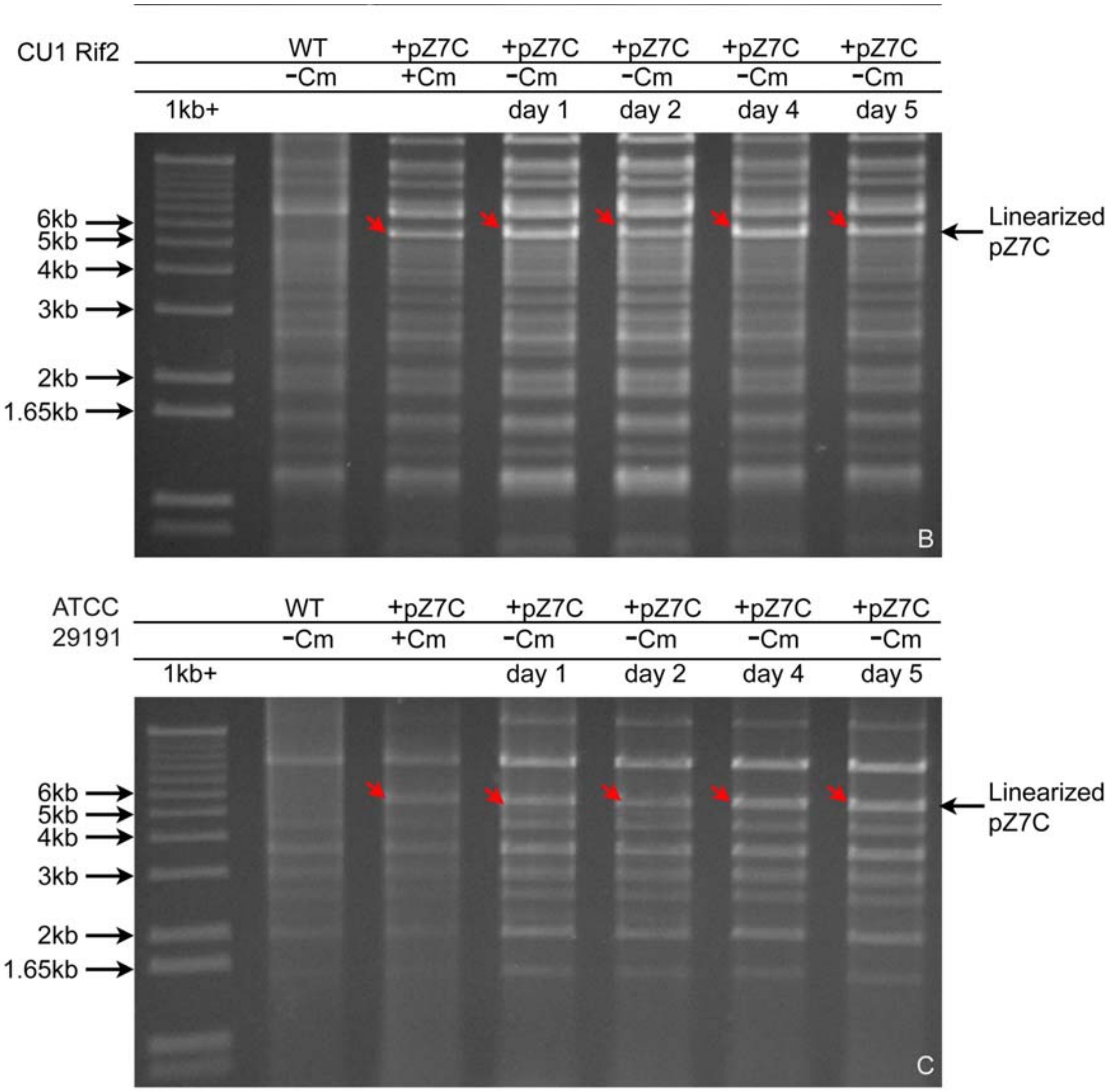

Supplement: Additional file 4 — Stability of pZ7C shuttle vector in Z. mobilis NCIMB 11163, CU1 Rif2 and ATCC 29191 strains cultured in media with/without selection agent. [file 1471-2180-14-68-S4.pdf]
