# Supplementary material for: pZMO7-Derived shuttle vectors for heterologous protein expression and proteomic applications in the ethanol-producing bacterium Zymomonas mobilis
Source: BMC Microbiol. 2014 Mar 15;14:68. doi: 10.1186/1471-2180-14-68 (PMC4004385; doi:10.1186/1471-2180-14-68)
Supplement: Additional file 5 — Quantitative-PCR determination of plasmid copy number for pZMO1A and pZMO7 in Z. mobilis NCIMB 11163 throughout the growth cycle. [file 1471-2180-14-68-S5.pdf]

Additional File 5

Quantitative-PCR determination of plasmid copy number for pZMO1A and pZMO7 in wild type *Z. mobilis* NCIMB 11163 throughout the growth cycle

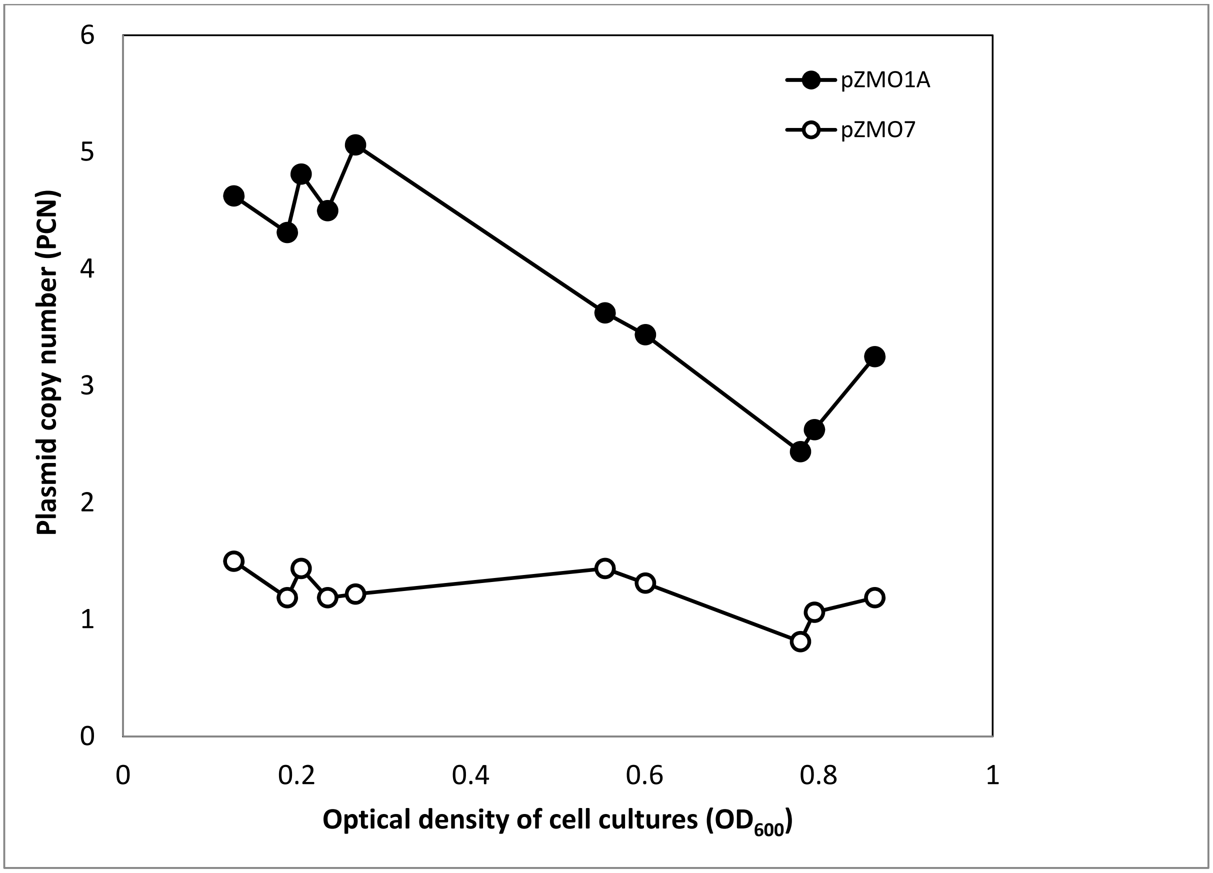

The plasmid copy numbers (PCN) of pZMO1A (filled circles) and pZMO7 (empty circles) during the static culture of *Z. mobilis* NCIMB 11163 cells in RM medium at 30°C, as determined using qPCR. Y-axis: plasmid copy number (PCN); x-axis: optical density of cell cultures ($OD_{600\ nm}$, absorbance units at 600 nm).
