# Supplementary material for: pZMO7-Derived shuttle vectors for heterologous protein expression and proteomic applications in the ethanol-producing bacterium Zymomonas mobilis
Source: BMC Microbiol. 2014 Mar 15;14:68. doi: 10.1186/1471-2180-14-68 (PMC4004385; doi:10.1186/1471-2180-14-68)
Supplement: Additional file 6 — Affinity-purification of recombinant GST protein expressed from plasmid pZ7-GST established in E. coli BL21 (DE3), and Z. mobilis ATCC 29191 and CU1 Rif2. [file 1471-2180-14-68-S6.pdf]

Additional Figure 6

Affinity-purification of recombinant GST protein expressed from plasmid pZ7-GST established in *E. coli* BL21 (DE3), *Z. mobilis* ATCC 29191 and CU1 Rif2

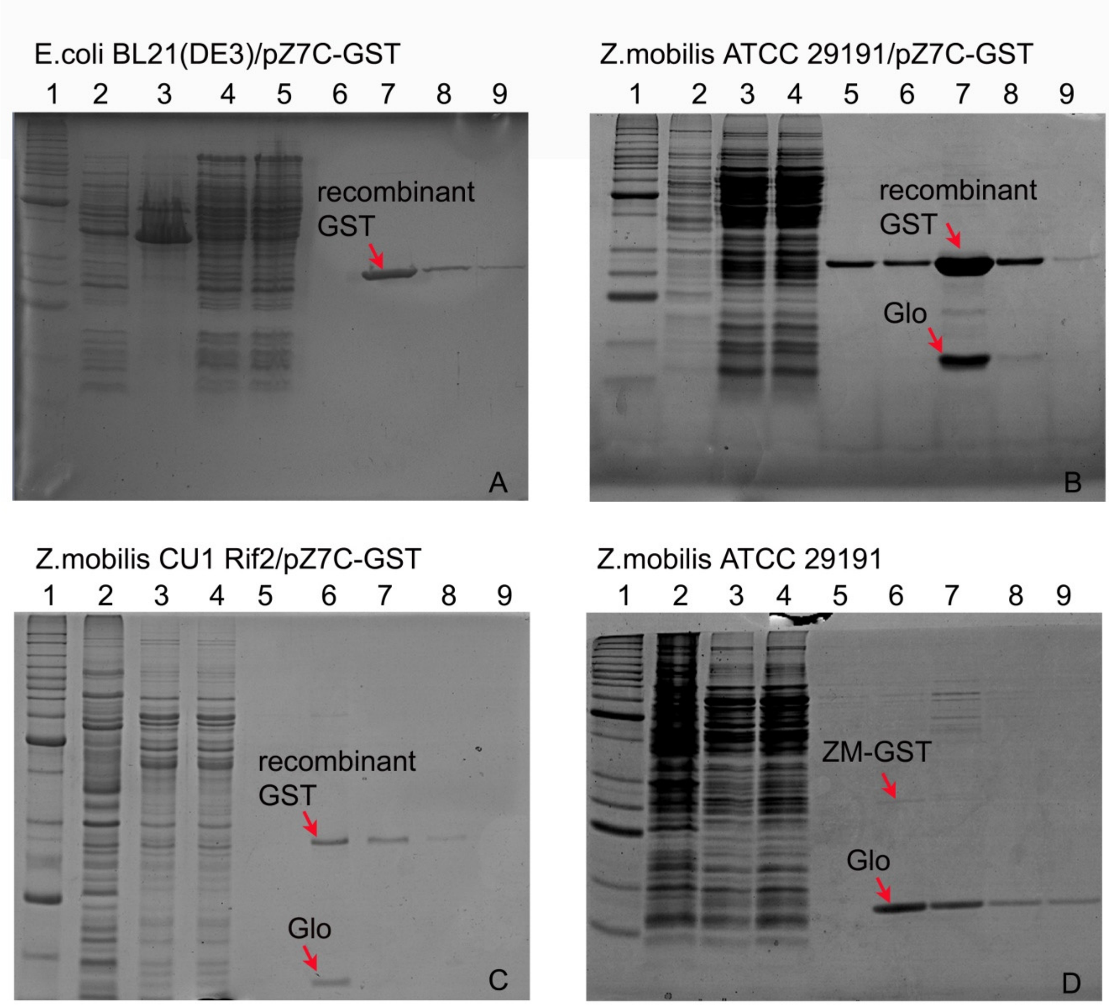

Panel A: *E. coli* BL21 (DE3)/pZ7-GST; **Panel B**: *Z. mobilis* ATCC 29191/pZ7-GST; **Panel C**: *Z. mobilis* CU1 Rif2/pZ7-GST; **Panel D**: wild type *Z. mobilis* ATCC 29191. All panels show analogous Coomassie Blue stained 15% SDS-polyacrylamide gels of proteins present in various cellular fractions, as well as in eluted fractions obtained after affinity chromatography purification using glutathione-sepharose resin. **Lane 1**: Benchmark protein ladder (Invitrogen); **lane 2**: cell debris after sonication; **lane 3**: supernatant of cell-free extract; **lane 4**: flow through from glutathione-sepharose affinity column; **lanes 5-9**: fractions 1-5 eluted with buffer containing 1mM glutathione. See methods section for detailed experimental procedures.
