# Supplementary material for: pZMO7-Derived shuttle vectors for heterologous protein expression and proteomic applications in the ethanol-producing bacterium Zymomonas mobilis
Source: BMC Microbiol. 2014 Mar 15;14:68. doi: 10.1186/1471-2180-14-68 (PMC4004385; doi:10.1186/1471-2180-14-68)
Supplement: Additional file 7 — Growth curves for wild type and pZ7C-GST plasmid-transformed Z. mobilis strains NCIMB 11163, CU1 Rif2 and ATCC 29191. [file 1471-2180-14-68-S7.pdf]

Additional File 7

Growth curves for wild type and pZ7C-GST plasmid-transformed *Z. mobilis* strains NCIMB 11163, CU1 Rif2 and ATCC 29191

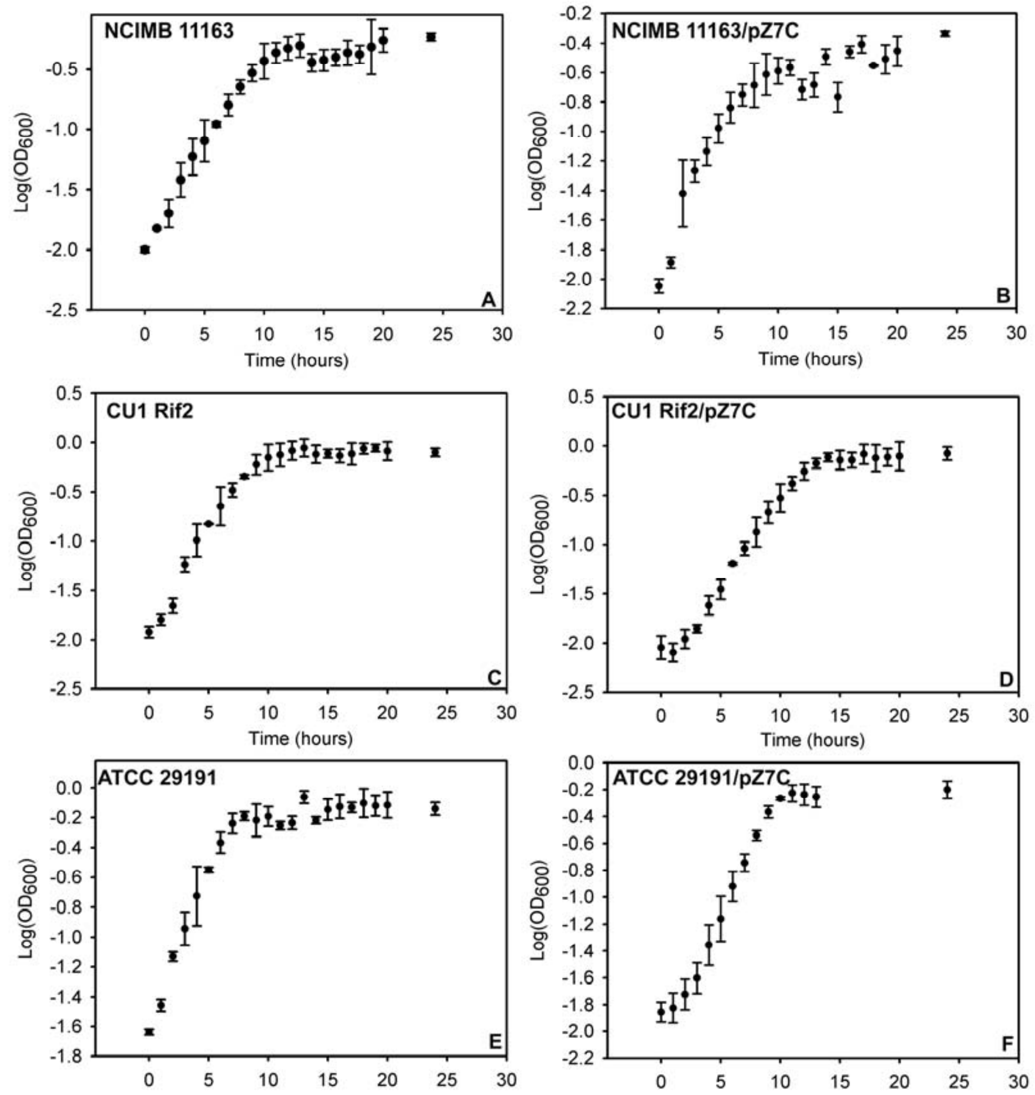

Panel A: Wild type *Z. mobilis* NCIMB 11163; **Panel B**: *Z. mobilis* 11163/pZ7-GST; **Panel C**: Wild type *Z. mobilis* CU1 Rif2; **Panel D**: *Z. mobilis* CU1 Rif2/pZ7C-GST; **Panel E**: Wild type *Z. mobilis* ATCC 29191; **Panel F**: *Z. mobilis* ATCC 29191/pZ7C-GST. All cultures were grown semi-aerobically without agitation at 30°C in RM media (wild type strains) or RM media containing 100μg/ml chloramphenicol (pZ7C-GST plasmid-transformed strains). Aliquots (1 ml) were withdrawn every 60 minutes for OD_{600nm} measurements. Experiments were performed in (at least) triplicate, with data plotted using the mean OD_{600nm} value ± standard deviation.
