# Supplementary material for: pZMO7-Derived shuttle vectors for heterologous protein expression and proteomic applications in the ethanol-producing bacterium Zymomonas mobilis
Source: BMC Microbiol. 2014 Mar 15;14:68. doi: 10.1186/1471-2180-14-68 (PMC4004385; doi:10.1186/1471-2180-14-68)
Supplement: Additional file 8 — Expression of GST-fusion proteins from respective pZ7-GST plasmid constructs established in E. coli. [file 1471-2180-14-68-S8.pdf]

Additional File 8

Expression of GST-fusion proteins from respective pZ7-GST plasmid constructs established in *E. coli*

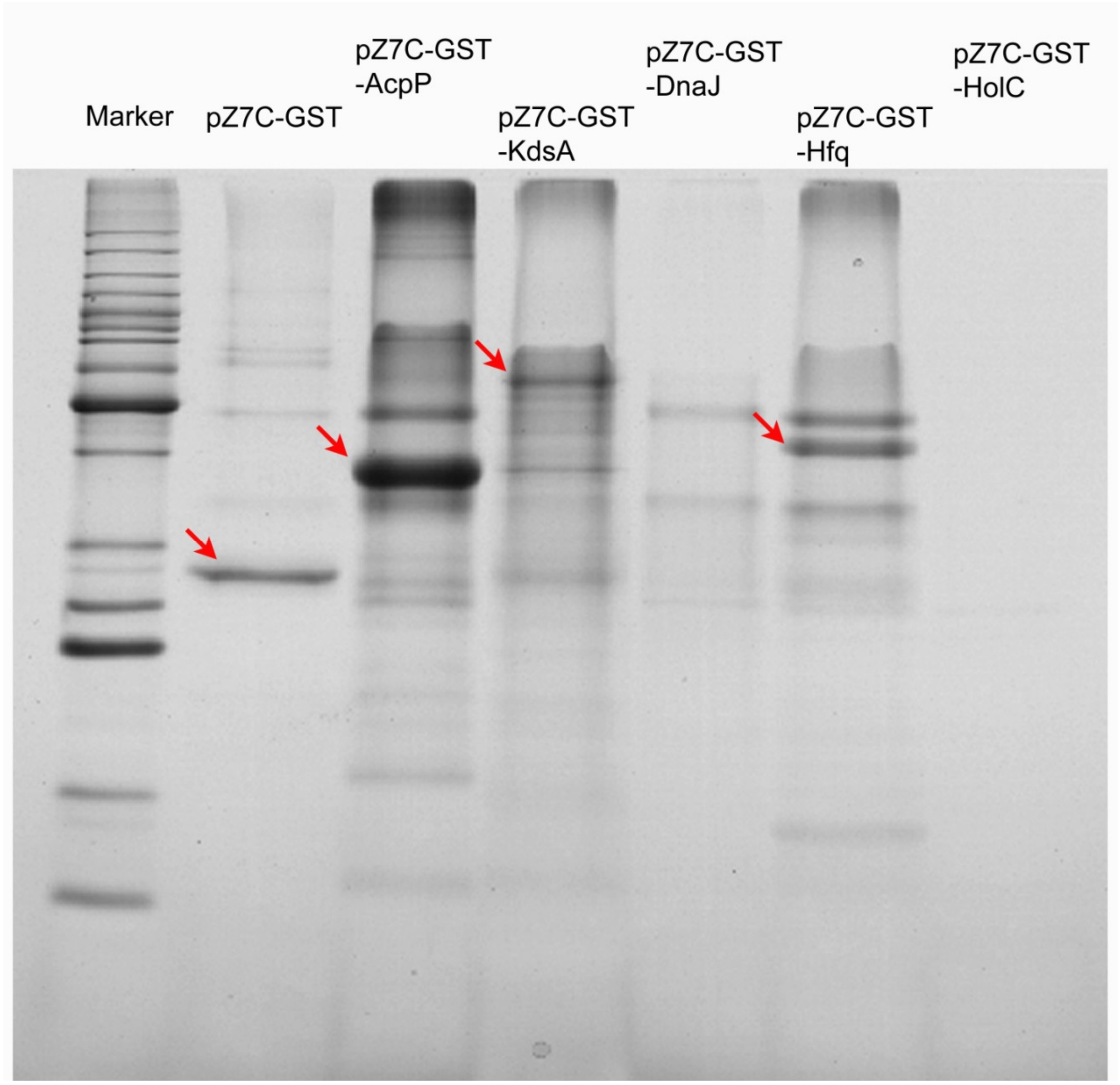

Image of a Coomassie Blue-stained 20% SDS-polyacrylamide gel showing eluents from GST-affinity column purifications of proteins from cell lysates prepared from *E. coli* BL21 (DE3) strains respectively containing pZ7-GST and the five pZ7C-GST-fusion protein plasmid constructs. From left to right: **lane 1**, benchmark protein ladder; **lane 2**, *E. coli*/pZ7C-GST; **lane 3**, *E. coli*/pZ7C-GST-AcpP; **lane 4**, *E. coli*/pZ7C-GST-KdsA; **lane 5**, *E. coli*/pZ7C-GST-DnaJ; **lane 6**, *E. coli*/pZ7C-GST-Hfq; **lane 7**, *E. coli*/pZ7C-GST-HolC. Red arrows indicate the positions of GST and the respective GST-fusion proteins. See Methods section for experimental details.
