# Supplementary material for: pZMO7-Derived shuttle vectors for heterologous protein expression and proteomic applications in the ethanol-producing bacterium Zymomonas mobilis
Source: BMC Microbiol. 2014 Mar 15;14:68. doi: 10.1186/1471-2180-14-68 (PMC4004385; doi:10.1186/1471-2180-14-68)
Supplement: Additional file 9 — Western blot analysis of pZ7C-GST fusion protein expression levels in Z. mobilis ATCC 29191 and CU1 Rif2. [file 1471-2180-14-68-S9.pdf]

Additional File 9

Western blot analysis of pZ7C-GST fusion protein expression levels in *Z. mobilis* ATCC 29191 and CU1 Rif2

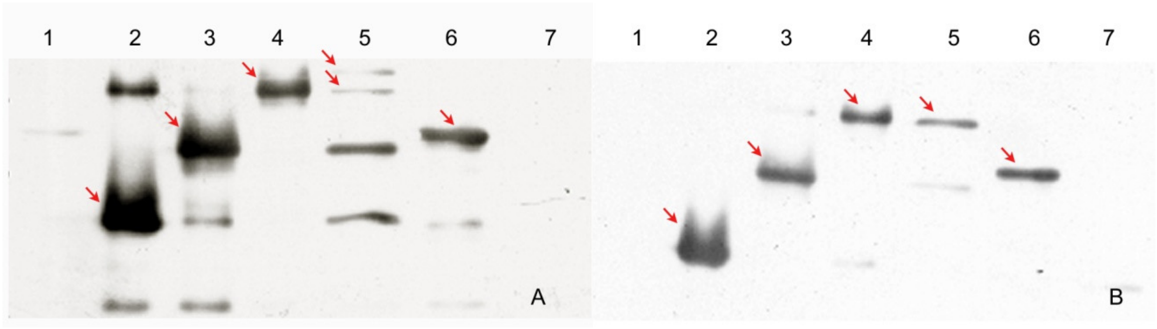

The expression patterns of the respective GST-fusion proteins encoded by the pZ7C-GST; pZ7C-GST-AcpP; pZ7C-GST-KdsA; pZ7C-GST-DnaJ; pZ7C-GST-Hfq; pZ7C-GST-HolC plasmids were analyzed in *Z. mobilis* ATCC 29191 and CU1 Rif2, using a Western blotting approach. Fractions eluted from GST-affinity column purifications of cell lysates prepared from strains containing the respective plasmid constructs were resolved on 20% polyacrylamide gels, and proteins were transferred to PVDF membranes. The membranes were probed using anti-GST primary antibodies, and visualized using a chemiluminescence approach. **Panel A**: *Z. mobilis* ATCC 29191, **Panel B**: *Z. mobilis* CU1 Rif2; lanes are equivalent in both gel blots. From left to right: **lane 1**, protein ladder; **lane 2**, pZ7C-GST; **lane 3**, pZ7C-GST-AcpP; **lane 4**, pZ7C-GST-KdsA; **lane 5**, pZ7C-GST-DnaJ; **lane 6**, pZ7C-GST-Hfq; **lane 7**, pZ7C-GST-HolC. Red arrows indicate the positions of recombinant GST and the respective GST-fusion proteins. N.B. two forms of the GST-DnaJ fusion protein are present in the *Z. mobilis* ATCC 29191/pZ7C-GST-DnaJ strain. See Methods section for experimental details.
